# The MCP-4/MCP-1 ratio in plasma is a candidate circadian biomarker for chronic post-traumatic stress disorder

**DOI:** 10.1038/tp.2016.285

**Published:** 2017-02-07

**Authors:** C Dalgard, O Eidelman, C Jozwik, C H Olsen, M Srivastava, R Biswas, Y Eudy, S W Rothwell, G P Mueller, P Yuan, W C Drevets, H K Manji, M Vythlingam, D S Charney, A Neumeister, R J Ursano, D M Jacobowitz, H B Pollard, O Bonne

**Affiliations:** 1 Department of Anatomy, Physiology and Genetics, Uniformed Services University School of Medicine, Uniformed Services University of the Health Sciences, Bethesda, MD, USA; 2 Collaborative Health Initiative Research Program (CHIRP), Uniformed Services University School of Medicine, Uniformed Services University of the Health Sciences, Bethesda, MD, USA; 3 Henry M. Jackson Foundation for Military Medicine, Bethesda, MD, USA; 4 Department of Preventive Medicine and Biometrics, Uniformed Services University School of Medicine, Uniformed Services University of the Health Sciences, Bethesda, MD, USA; 5 Mood and Anxiety Disorders Branch, National Institute for Mental Health, Bethesda, MD, USA; 6 Janssen Research & Development, LLC, of Johnson and Johnson, Titusville, NJ, USA; 7 United States Department of Defense, Washington, DC, USA; 8 Office of the Dean, Mount Sinai Medical School, New York, NY, USA; 9 Mitsubishi Tanabe Pharma Development America, Jersey City, NJ, USA; 10 Department of Psychiatry, Uniformed Services University School of Medicine, Uniformed Services University of the Health Sciences, Bethesda, MD, USA; 11 Center for the Study of Traumatic Stress, Uniformed Services University School of Medicine, Uniformed Services University of the Health Sciences, Bethesda, MD, USA; 12 Department of Psychiatry, Hadassah Medical Center, Jerusalem, Israel

## Abstract

Post-traumatic stress disorder (PTSD) is psychiatric disease, which can occur following exposure to traumatic events. PTSD may be acute or chronic, and can have a waxing and waning course of symptoms. It has been hypothesized that proinflammatory cytokines and chemokines in the cerebrospinal fluid (CSF) or plasma might be mediators of the psychophysiological mechanisms relating a history of trauma exposure to changes in behavior and mental health disorders, and medical morbidity. Here we test the cytokine/chemokine hypothesis for PTSD by examining levels of 17 classical cytokines and chemokines in CSF, sampled at 0900 hours, and in plasma sampled hourly for 24 h. The PTSD and healthy control patients are from the NIMH Chronic PTSD and healthy control cohort, initially described by Bonne *et al.* (2011), in which the PTSD patients have relatively low comorbidity for major depressive disorder (MDD), drug or alcohol use. We find that in plasma, but not CSF, the bivariate MCP4 (CCL13)/ MCP1(CCL2) ratio is ca. twofold elevated in PTSD patients compared with healthy controls. The MCP-4/MCP-1 ratio is invariant over circadian time, and is independent of gender, body mass index or the age at which the trauma was suffered. By contrast, MIP-1β is a candidate biomarker for PTSD only in females, whereas TARC is a candidate biomarker for PTSD only in males. It remains to be discovered whether these disease-specific differences in circadian expression for these specific immune signaling molecules are biomarkers, surrogates, or drivers for PTSD, or whether any of these analytes could contribute to therapy.

## Introduction

Post-traumatic stress disorder (PTSD) is a psychiatric disease that may occur following exposure to traumatic events.^1^ PTSD may be acute or chronic, and generally follows a waxing and waning course of symptoms that can persist for months, years or decades^2^ (http://www.behavenet.com/capsules/disorders/ptsd.htm). The disorder is nearly always accompanied by profound difficulties in sleep.^3, 4, 5^ Some studies found chronic PTSD patients to have intrinsically low levels of circulating cortisol,^6, 7^ suggesting a possible failure of feedback inhibition, leading to excessive levels of cell-mediated and proinflammatory cytokine expression.^8, 9, 10^ A recent candidate gene study showed that upstream drivers for PTSD include TNFα and the glucocorticoid receptor.^11^ Consistently, a recent study of plasma, taken hourly between 2000 and 0700 hours, showed that in civilian patients with chronic PTSD, cortisol levels are lower than in controls at the diurnal nadir, and shifted forward by ca. 2 h with rise towards a zenith cortisol levels merge at 0500 hours (Supplementary Figure 1).^7^ The cortisol rise time is further delayed if the PTSD patients are also comorbid with major depressive disorder (MDD).^7^

Circulating levels of immune cells, including monocytes, neutrophils and lymphocytes are regulated in a circadian manner, as are circulating levels of many of the cytokines and chemokines secreted by these and related cells.^12^ However, comprehensive circadian studies in PTSD patients of either immune cells, or circulating immune mediators, are not yet available. Nonetheless, further implicating immune cell dysfunction in PTSD are studies showing suppressed gene expression in purified monocytes from blood samples, selected from a randomly chosen cohort of chronic PTSD patients from San Francisco.^13^ Similar conclusions have been drawn from studies of peripheral blood mononuclear cells, from blood samples from chronic PTSD cohorts of either holocaust survivors,^14^ or World Trade Center survivors.^15^ These data thus suggest that an experimental focus on immune cell mediators in PTSD plasma might provide robust biomarkers for this disorder.

Complicating this line of analysis, however, are reports that while PTSD patients who are co-morbid *with* MDD do have higher levels of serum interleukin-6 (IL-6) in samples taken at unspecified times,^16^ those PTSD patients without co-morbid MDD have serum IL-6 levels that are virtually identical to levels in normal controls.^16^ In addition, in a carefully controlled study of cerebrospinal fluid (CSF) from an NIMH cohort of civilian chronic PTSD patients relatively free from MDD, concentrations of Corticotrophin Releasing Factor (CRF), IL-6, brain-derived neurotrophic factor, IGF-1 and Substance P were found to be identical to levels in CSF from healthy controls.^1^ These results thus cast doubt on the involvement of some immune mediators in PTSD patients with limited comorbidities.

However, the diurnal variation in differences of cortisol levels between PTSD and healthy subjects described by Vythilingam *et al.*^7^, as well as concurrent ACTH differences (Supplementary Figure 1b), compelled us to hypothesize that a more comprehensive analysis of circulating immune mediators, many known to be under circadian control, might identify downstream circulating biomarkers for PTSD. In the current study we performed a preliminary analysis of plasma samples collected at 0200 and 0900 hours. We found that out of 17 different cytokines and chemokines, elevated chemokine MCP-4 (monocyte chemoattractant protein-4; CCL11) and reduced chemokine MCP-1 (monocyte chemoattractant protein-1; CCL2) appeared to significantly distinguish PTSD subjects from healthy controls. On the basis of these findings we further tested: (1) whether a complete circadian analysis of plasma MCP-4 or MCP-1 might identify a PTSD-specific biomarker: the MCP-4/MCP-1 ratio was found to be a significantly elevated, gender-independent, biomarker for PTSD over the entire circadian period; (2) whether any chemokines might distinguish PTSD patients from healthy controls on the basis of gender over the circadian period: we found MCP-4 and MIP-1β (monocyte interacting protein 1βCCL4) to significantly biomark PTSD in females, whereas MCP-1 and the lymphokine TARC (thymus and activation-regulated chemokine; CCL17) significantly biomarks PTSD in males; and (3) whether chemokine or cytokines in CSF, taken at 0900 hours, distinguished between PTSD patients and healthy controls: we found that they did not, consistent with the results of Bonne *et al.*^1^ We conclude that although the MCP-4/MCP-1 ratio appears to be a compelling candidate biomarker for PTSD, it remains to be discovered whether the processes affecting chemokine expression in PTSD have a mechanistic relationship to intrinsic biological, behavioral or structural deficits in the PTSD brain, and whether any of these analytes could also be candidate targets for therapy.

## Materials and methods

### Patients

Sixteen medication-free outpatients with chronic civilian PTSD (median age 31.5 years, 11 women/5 men) and 11 non-traumatized, healthy subjects (median age 29.5 years, 9 women, 5 men) matched to PTSD patients with respect to age, sex and body mass index (BMI), selected on the basis of availability of sufficient hourly plasmas and CSF, from the Bonne *et al.*^1^ cohort, participated in the study (Supplementary Table 1). Time elapsed from trauma exposure was 26±14 years in pre-pubertal trauma, and 10.5±10.5 years in adult exposure. Patients were physically healthy, had received no psychotropic medications for at least 3 weeks before the lumbar puncture (6 weeks for fluoxetine) and did not meet criteria for alcohol or substance abuse or dependence for at least 6 months prior to the study. All participants gave informed consent to take part in the study, using procedures established by the National Institutes of Health Institutional Review Board (NIMH # 02-M-0317).

### Psychiatric diagnoses

Psychiatric diagnoses were established using the Structured Clinical Interview for DSM-IV. The severity of PTSD was determined using the Clinician-Administered PTSD Scale (CAPS). Severity of depressive, anxiety and overall symptoms was assessed using the Inventory of Depressive Symptomatology (IDS), Hamilton Anxiety Rating Scale (HAMA) and Clinical Global Impression - Severity scale (CGI-S), respectively. Individuals with PTSD and controls did not differ with regard to age, gender distribution, race or BMI. Severity of PTSD was moderate, with a median CAPS score of 70 (range 54–95). Depression (IDS 16.4±8.2), Anxiety (HAMA 13.1±6.8) and overall symptom severity levels (CGI-S 4±1.2) were moderate as well.

### Lumbar pucture and plasma collection

Participants were studied at the National Institutes of Health, under an institutional review board approved protocol. Lumbar puncture was performed between 0800 and 0900 hours by an experienced physician. A 20-gauge introducer needle was inserted and ~15 cc of CSF was withdrawn, centrifuged at 4000 r.p.m., and frozen in aliquots at −80°C for later assay. Four to 6 days after the procedure, participants had 27 hourly plasma sample drawings, beginning at 0700 hours and ending at 0900 hours the following day. Individuals were implanted with indwelling intravenous catheters. Nighttime sampling was done without disturbing participants. All blood samples were immediately anti-coagulated with sodium citrate, and the plasma collected by centrifugation. Following immediate centrifugation, the supernatant solutions were aliquoted, stored frozen at −80 °C, and reserved for later analysis.

### Analysis of cytokines and chemokines

Two multiplexed assays for cytokines and chemokines were used for analysis of patient and control plasma samples on the SECTOR Imager 6000 instrument (MesoScale Discovery, Gaithersburg, MD, USA) The first of these assays was the Human ProInflammatory 9-Plex Assay for the measurement of IL-2, IL-8, IL-12p70, IL-1β, GM-CSF, IFN-γ, IL-6, IL-10 and TNF-α (MesoScale catalog #K15007C-4). The second of these assays was the Human Chemokine 9-Plex Assay for the measurement of Eotaxin 1, MIP-1β, Eotaxin-3, TARC, IP-10, IL-8, MCP-1 (CCL2), MDC and MCP-4 (CCL13) (catalog #K15001C-1). The samples were added to plates that were pre-coated with capture antibodies for the specific cytokines. The plates was sealed and shaken at room temperature for 2 h. The plates were washed in PBS+0.05% Tween-20 and detection antibody solution (1 × or 1 μg ml^−1^) was then added. The plates were once again sealed and set to shake at room temperature for 2 h. The plate was then washed once more in PBS+0.05% Tween-20. Read buffer was added at a 2 × concentration and the plate was read on the SECTOR 6000 Imager. Recently, the Human Chemokine 9-plex has been replaced with new reagents by the V-Plex. However, these studies were completed using the original Human Chemokine 9-plex, available (and recommended) by special order from the company.

### Bioinformatics

#### Statistics

Accuracy of analyte measurements were assessed on the basis of reproducible technical replicates, low percentage coefficient of variation (CV<5%), presence within the linear portion of the standard curve, and a value above the lower limit of detection (LLOD). The differences between PTSD samples and normal controls were calculated using a two-tailed *t*-test to calculate a *P*-value. The 0900 hours CSF analysis of 17 analytes used the stringent Bonferroni multiple correction for significance (viz., *P*<0.003, rather than *P*<0.05). The 9-plex chemokine analysis for circadian plasma studies at 0900 and 0200 hours used the Bonferroni multiple correction criterion for significance (viz.*,* *P*<0.005, rather than *P*<0.05). The Bonferroni multiple comparisons correction was not applied to 27-h circadian sampling, inasmuch as measurements were not independent, and provided that the proper levels of significance were attained at most, if not all, of the time points. Values for all analytes, measured at 0900 hours, for healthy controls, were mostly found to be statistically indistinguishable from data published by others who had also used the industry-standard MesoScale electrochemiluminescence Sector 6000 platform for analysis.^17, 18, 19, 20^ Times were standardized to the sunrise time on the day blood sampling began (*Z*=0, the Zeitgeber). Circadian rhythms were modeled by fitting data to a cosine function.

We also conducted tests to check for equality of the variances of the amounts of each analyte in the two groups, PTSD and healthy controls. When we applied these tests to the circadian changes in analyte serum levels, the variances were found to change by more than two fold over the 26 h study. Moreover, at different times the two groups oscillated as to which one had the larger variance. We attribute these changes in variance over circadian time to the fact that when the level of an analyte is at a plateau the variance reflects the intrinsic heterogeneity of the cohort. However, when the level starts to rise, or decline, each individual in the cohort starts at a slightly different time. Thus, the specific variances for both patients and healthy controls reflect both the heterogeneity of the individual average levels as well as the heterogeneity of the individual small time shifts. Thus, for those analytes where we find shifts in the time course between the patients and the control groups, the size as well as the order of the variances do change with time.

#### Mixed model statistical analysis

Data were analyzed using a mixed model for repeated measures with group as a between-subjects factor, zeit as a within-subjects factor, and a subject random effect. Gender, BMI and age at trauma were included as covariates in some models.This approach allows for within-subject correlation without requiring complete data from every subject. Models were estimated using the MIXED procedure in SPSS version 22 (IBM, Armonk, NY, USA).

#### Zeitgeber correction and interpolation

The actual sunrise time on the date of sampling was used to convert the clock-time (T) of each sample withdrawal to the time relative to sunrise (*Z*). The levels of each analyte were linearly interpolated between consecutive samples at whole 15 min Z intervals. In order to average results from multiple subjects, the interpolated analyte levels were converted to logarithmic values before averaging. Samples drawn more than 12 h after sunrise on the day of sampling are plotted as negative *Z* value, that is, at the respective time prior to the next day sunrise.

#### Logarithmic slopes

For each sampling the logarithmic slope was estimated by calculating a 3 data point regression line consisting of the immediate point, the previous point, and the next point. The slopes were then smoothed using a 3 point running window averaging, so that the smoothed logarithmic slope is defined by 5 data points. These slopes were then interpolated at 15 min intervals and averaged across the PTSD and Control groups.

#### Zeniths (peaks) and nadirs (troughs)

Zeniths were defined as periods of near zero slope preceded by a period of high positive slope, and followed by a period of high negative slope. This definition allows for a broad peak and avoids local maxima which might otherwise be randomly influenced by noise. Nadirs were oppositely defined.

## Results

### Measurement of cytokines and chemokines in PTSD plasma at 0900 hours local time

On the basis of the fact that sampling of blood in a clinical setting would most likely occur in the morning, and in accordance with the time of the lumbar puncture, we initially analyzed the 0900 hours samples of plasma, collected according to local time. Table 1 shows measurements of cytokines and chemokines in plasma collected from both PTSD and healthy control patients at the 0900 hours time point. In this case, MCP-4 is significantly elevated by ca. 43%. The *P-*value is 0.01 and the area under the curve (AUC) is 0.82. By contrast, MCP-1 is reduced by ca. 20%. However, although the *P-*value for MCP-1 fails the significance test, the AUC value, 0.82, is the highest of all analytes. The MCP-1 and MCP-4 data thus stratify in opposite directions. We therefore tested the possibility that a significant multiparameter biomarker could be developed for PTSD from a ratio of the two values. Importantly, this strategy for disease-specific biomarker discovery has had recent successful precedence for Alzheimer disease^21^ and for temporal lobe epilepsy.^17^ As shown in the last row in Table 1, by dividing these two inversely directional classifiers, the MCP-4/MCP-1 ratio at 0900 hours (local time) is elevated 84% in PTSD plasma, and provides a highly significant candidate metric for PTSD plasma collected at 0900 hours. Importantly, the ratio of analyte concentrations, is independent of hemo-concentration. The difference is significant, based on both a low *P-*value of 0.004, which survives a Bonferroni correction, and a high AUC value of 0.84. This PTSD-specific metric has a higher value at 0900 hours than 0200 hours (see below), indicating that the signal may be diurnal.

Several other individual cytokines and chemokines were also found to be significantly different in the PTSD 0900 hours plasmas compared with healthy control plasmas. These included IL-1β (reduced more than twofold; *P*=0.04; AUC=0.71); TNFα (elevated ca. 64% *P*=0.03; AUC=0.76); and IP-10 (elevated ca. 50% *P*=0.04; AUC=0.73. Nonetheless, while these differences are significant at the 0.05 level (but not after accounting for multiple comparisons), they are relatively modest in effect size, and AUC values calculated from the receiver operation condition (ROC) curves are modest as well. Furthermore, our attempts to create candidate inversely directional classifiers from these elevated analytes with reduced MCP-1 resulted in less significant metrics than the one calculated from the MCP-4/MCP-1 ratio. Thus the MCP-4/MCP-1 ratio seems to be the best candidate biomarker for PTSD in the 0900 hours plasma.

### Measurement of cytokines and chemokines in PTSD plasma at 0200 hours

Supplementary Table 2 shows measurements of cytokines and chemokines in plasma collected from both PTSD and healthy control patients at 0200 hours. Although there are differences in expression levels for multiple analytes, none of them individually rise to the level of significance based on *P-*value alone. However, the table indicates that two analytes, MCP-1 and MCP-4, still vary in opposite directions, and each has among the highest AUC values on the list. For the case of PTSD, the last row in Supplementary Table 2 shows that the MCP-4/MCP-1 ratio is 34% elevated in 0200 hours PTSD plasma, compared with healthy controls. The *P-*value, based on a two-tailed t-test, is 0.02, indicating significance, and the ROC curve has an AUC of 0.75. This multiparameter analysis thus also identifies the MCP-4/MCP-1 ratio as a candidate binary classifier for PTSD compared to healthy controls in both 0900 and 0200 hours plasma samples.

### Effect of PTSD on the circadian profile for the plasma MCP-4/MCP-1 ratio, and on individual plasma chemokines

To test for PTSD-specific circadian changes in MCP-4, MCP-1 and the MCP-4/MCP-1 ratio, and 7 individual chemokines in plasma we analyzed a 24 h profile discovery fraction from 5 PTSD and 5 healthy control patients (Supplementary Figure 2a). The data indicate that across the entire 24 h time period, the scale-free MCP-4/MCP-1 ratio for PTSD patients remains approximately twice that of healthy control patients. Supplementary Figure 2b shows that the MCP-4/MCP-1 ratio for PTSD patients is significantly different from the healthy controls across the entire circadian period. Thus the ratio itself might constitute a metric valid for any blood collection. In this limited subset, MCP-4 is alone significantly sensitive to the PTSD condition, while the other chemokines are not significantly different.

**Figure 1:**
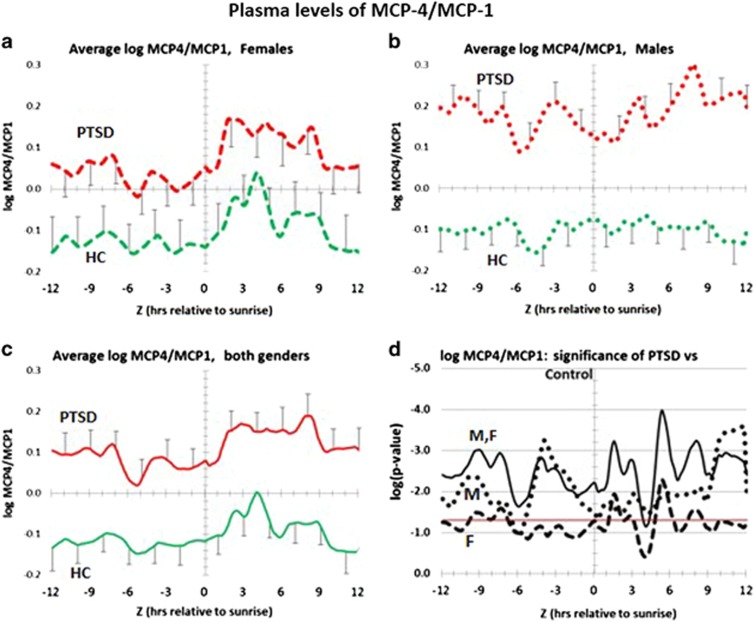
Distribution of MCP-4/MCP-1 ratio in plasma over a circadian interval for patients with PTSD and healthy controls. (**a**) Differences between average log plasma MCP-4/MCP-1 ratio in female PTSD patients vs female healthy controls. Error bars are ±s.e.m. *P-*values for the difference at each hour are shown in **d**. PTSD females are greater than healthy control females at every hour and the differences are significant, or trend to being significant at each hour (*P*⩽0.05), across circadian time. (**b**) Differences between average log plasma MCP-4/MCP-1 ratio in male PTSD patients vs male healthy controls. Error bars are ±s.e.m. *P*⩽values for the difference at each hour are shown in **d**. PTSD males are significantly greater than healthy control males at every hour (*P*⩽0.05). (**c**) Differences between average log plasma MCP-4/MCP-1 ratio in all PTSD patients vs all healthy controls. Error bars are ±s.e.m. *P-*values for the difference at each hour are shown in **d**. All PTSD patients are greater than all healthy controls at every hour and the differences are significant (*P*⩽0.05). This significance is based on common differences between both male and female PTSD patients relative to male and female healthy controls. (**d**) Significance of log plasma concentration differences for MCP-4/MCP-1 ratio over circadian time. Vertical axis is log *P-*value. Red horizontal line is the same as *P*=0.05 on a arithmetic scale. Male PTSD (dotted black line); female PTSD (dashed black line); all PTSD (solid black line). All PTSD patients have a significantly higher MCP-4 /MCP-1 ratios than all healthy controls. HC, healthy controls; PTSD, post-traumatic stress disorder.

To validate this discovery result, we performed the same circadian analysis on the *complete* collection of plasma samples from the PTSD and healthy control cohorts. Inasmuch as females have been reported to be more likely to suffer from PTSD than males,^22, 23^ we also tested our population for gender effects. Figure 1a and Figure 1b show that the MCP-4/MCP-1 ratio for PTSD is significantly higher than healthy controls for both female and male PTSD patients. Consistently, Figure 1c shows that significant elevation of the MCP-4/MCP-4 ratio is observed when both male and female PTSD patients are grouped together. Statistically, Figure 1d shows that for both genders, the *P-*values are ca. ⩽0.05 over the entire circadian time period. Furthermore, by pooling all PTSD patients and comparing them with all healthy controls, the *P-*values for each time point across circadian time is ca. 0.001. This difference is significant, across the entire circadian cycle, even after correcting for nine multiple comparisons.

**Figure 2:**
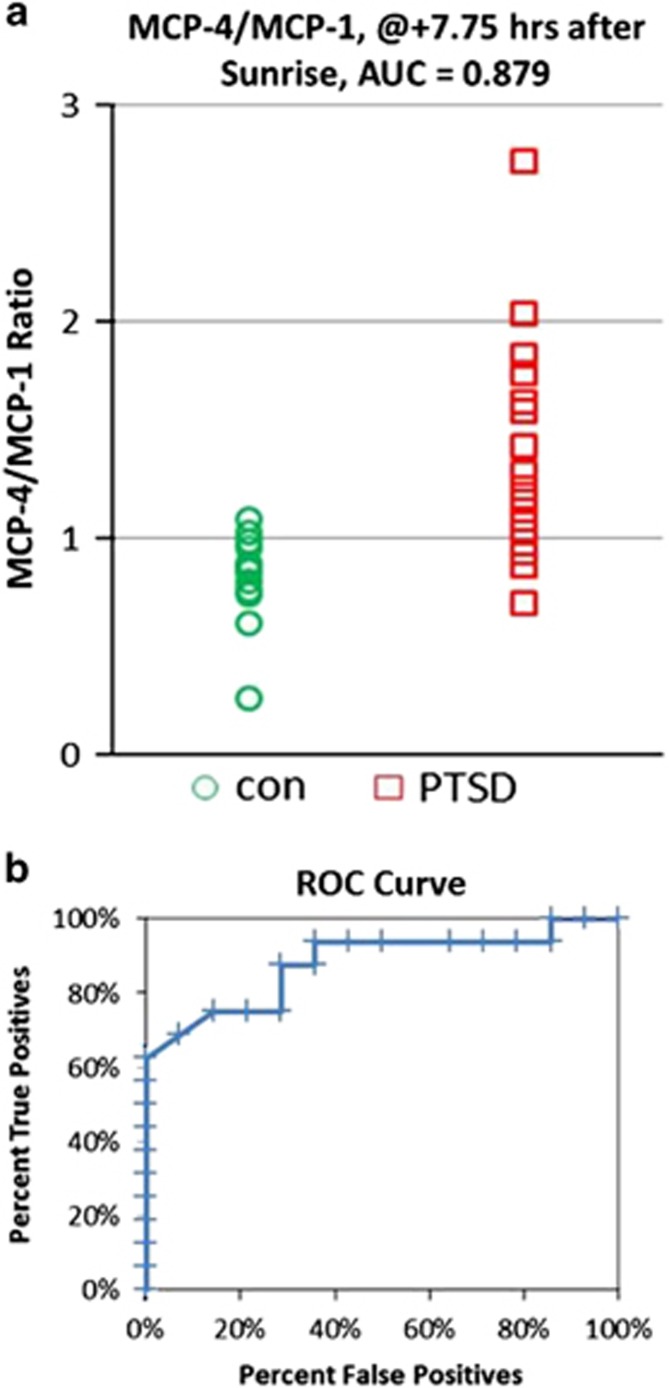
Dot-plot for MCP-4/MCP-1 ratio in patients with post-traumatic stress disorder (PTSD) and healthy controls at 7.75 h after sunrise (*Z*). (**a**) Ratios of MCP-4/MCP-1 for each PTSD patient and healthy control at *Z*+7.75 h. Color code: Green=healthy controls; Red=PTSD patients. Both genders are included. The difference is significant. (**b**) Receiver operating condition (ROC) curve. Area under the curve (AUC) is 0.879.

To further test the possibility that the MCP-4/MCP-1 ratio might be clinically useful for PTSD diagnosis, we compared the composite data as a dot-plot, and calculated a ROC curve. Figure 2a shows that when the ratio is plotted at *Z*=7.75 h, the difference is statistically significant (*P*<0.001) even after accounting for multiple comparisons of nine chemokines. It is worth noting that there are also multiple time point comparisons performed. But since the distribution of p-values is such that each one of them is at, or close to, the Bonferroni criterion, there is no need to further account for them. Furthermore, when ratios at *Z*=7.75 are plotted as a ROC, the AUC is 0.879 (Figure 2b). These data are therefore consistent with the possibility that the MCP-4/MCP-1 ratio might be a biomarker for PTSD, and that this candidate diagnostic biomarker might be valid for both male and female PTSD patients.

Finally, to test for possible entrainment differences for the MCP-4/MCP-1 ratio between PTSD and healthy control populations across circadian time, we calculated the percent of individual subjects within each population who were above their own daily averages. Entrainment refers to the alignment of the circadian system's period and phase to the period and phase of an external rhythm. In this case the external rhythm is defined by time between sunrise times. Supplementary Figure 3 shows these entrainment differences for females (Supplementary Figure 3a), males (Supplementary Figure 3b) and both genders (Supplementary Figure 3c). Both PTSD and healthy controls peak after sunrise. However, ~70% of female PTSD patients and 80% of male PTSD patients show extended elevation of the MCP4/MCP-1 ratio beyond time *Z*=6 h. However, based on *P-*values, Supplementary Figure 3d shows that these differences only trend towards significance.

### Gender-dependent effect of PTSD on the circadian profiles for MCP-4 and MCP-1

**Figure 3:**
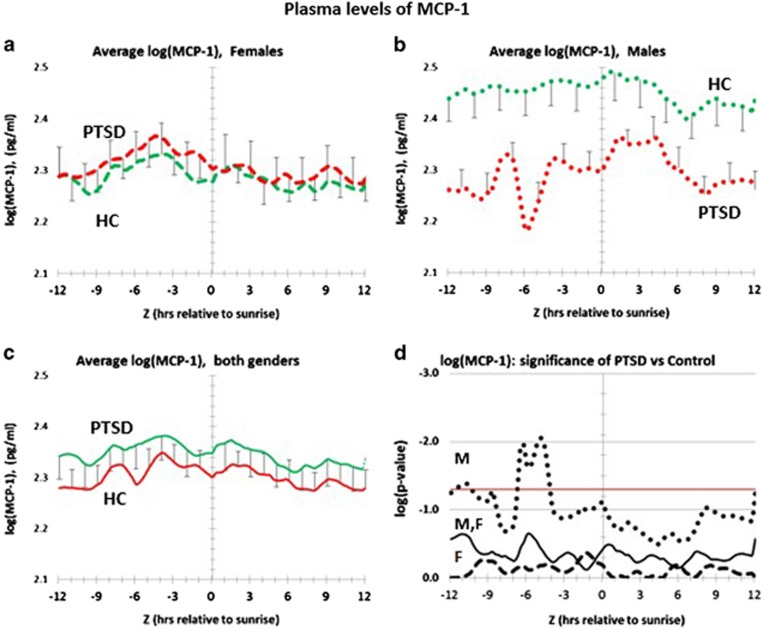
Distribution of plasma levels of MCP-1 over a circadian interval for patients with post-traumatic stress disorder (PTSD) and healthy controls (HC). (**a**) Differences between average log plasma MCP-1 levels in female PTSD patients vs female healthy controls. Error bars are ±s.e.m. Mean levels for PTSD Females are not significantly different from healthy control females at every hour, but the differences are not significant (**d**). (**b**) Differences between average log plasma MCP-1 levels in male PTSD patients vs male healthy controls. Error bars are ±s.e.m. Mean levels for PTSD males are lower than healthy control males at every hour, but the differences only trend towards significance, except in the time interval of (−6>*Z*>−4) h (**d**). (**c**) Differences between average log plasma MCP-1 levels in all PTSD patients vs all healthy controls. Error bars are ±s.e.m. *P-*values for the difference at each hour are shown in **d**. Mean values for all PTSD patients trend lower than mean values for all healthy controls (**d**). (**d**) Significance of log plasma concentration differences for MCP-1 over circadian time. Vertical axis is log *P-*value. Red horizontal line marks *P*=0.05 on a logarithmic scale. Male PTSD (dotted black line); female PTSD (dashed black line); all PTSD (solid black line). The only significant difference is for male PTSD patients in the time interval (−6>*Z*>−4) h.

Having validated the value of the MCP-4/MCP-1 ratio as a gender-independent candidate biomarker for PTSD, we next investigated the extent to which any of the individual chemokines might be gender-dependent biomarkers for PTSD. Figure 3 shows the complete circadian profile for MCP-1. As shown in Figures 3a and b in plasma from female and male PTSD, respectively, MCP-1 levels trend lower in males, but not in females. For both genders the trend is to be lower in PTSD (see Figure 3c, and statistics in Figure 3d.). Supplementary Figure 4 tests for entrainment differences between PTSD and healthy controls for females (Supplementary Figure 4a), males (Supplementary Figure 4b), and both genders (Supplementary Figure 4c). Both PTSD and healthy controls peak before sunrise. MCP-1 Entrainment differences between PTSD and healthy controls are only significant for males at ca. (*Z*−12) h (Supplementary Figure 4d). Early morning peaks for MCP-1 are consistent with previous data for otherwise healthy controls.^24, 25^

**Figure 4:**
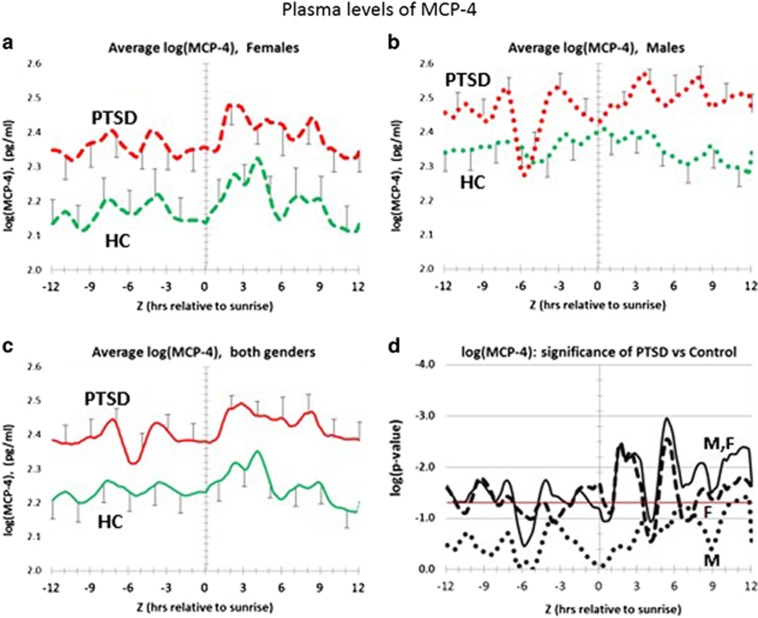
Distribution of plasma levels of MCP-4 over a circadian interval for patients with post-traumatic stress disorder (PTSD) and healthy controls. (**a**) Differences between average log plasma MCP-4 levels in female PTSD patients vs female healthy controls. Error bars are ±s.e.m. *P*-values for the difference at each hour are shown in **d**. PTSD Females are greater than healthy control females at every hour and the differences are significant (*P⩽*0.05). (**b**) Differences between average log plasma MCP-4 levels in male PTSD patients vs male healthy controls. Error bars are ±s.e.m. *P-*values for the difference at each hour are shown in **d**. PTSD males only trend greater than healthy control males at every hour, except in the interval (+9<*Z*<+12) h, where the differences are significant (*P⩽*0.05). (**c**) Differences between average log plasma MCP-4 levels in all PTSD patients vs all healthy controls. Error bars are ±s.e.m. *P-*values for the difference at each hour are shown in **d**. All PTSD patients are greater than all healthy controls at every hour and the differences are significant (*P⩽*0.05). However, this significance is based on preponderance of female PTSD relative to male PTSD patients. (**d**) Significance of log plasma concentration differences for MCP-4 over circadian time. Vertical axis is log *P-*value. Red horizontal line marks *P*=0.05 on a logarithmic scale. Male PTSD (dotted black line); female PTSD (dashed black line); all PTSD (solid black line). Female PTSD patients have a significantly higher MCP-4 expression than female healthy controls. Significance of both genders (M,F) is based on preponderance of female PTSD relative to male PTSD patients.

Figure 4 shows the complete circadian profile for MCP-4. As shown in Figures 4a and b in plasma from female and male PTSD, respectively, MCP-4 levels are higher across all circadian time. However, although PTSD females are significantly different from healthy control females, Figure 4d shows that male PTSD patients only trend towards significance. Supplementary Figure 5 tests for entrainment differences for MCP-4 between PTSD and healthy controls. The data show that ca. 80% of PTSD and healthy controls peak after sunrise. Entrainment differences for females (Supplementary Figure 5a) and males (Supplementary Figure 5b) can be seen at (6<*Z*<12) h. However, Supplementary Figure 5d shows that these differences only trend towards significance, except for male PTSD patients at (9<*Z*<12) hours.

### Gender-dependent effect of PTSD on the circadian profile for plasma MIP-1β

**Figure 5:**
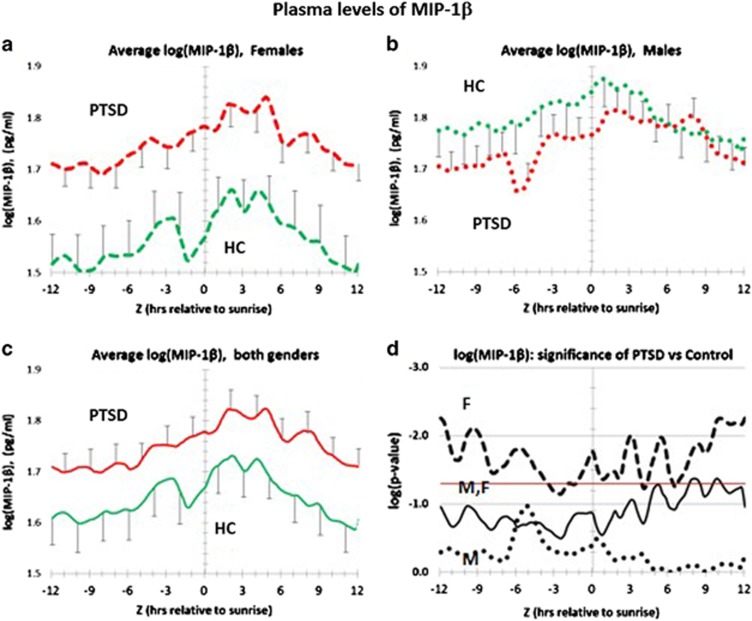
Distribution of plasma levels of MIP-1β over a circadian interval for patients with post-traumatic stress disorder (PTSD) and healthy controls (HC). (**a**) Differences between average log plasma MIP-1β levels in female PTSD patients vs female healthy controls. Error bars are ±s.e.m. *P-*values for the difference at each hour are shown in **d**. PTSD Females are greater than healthy control females at every hour and the differences are significant, or trend to being significant at each hour across circadian time. (*P⩽*0.05). (**b**) Differences between average log plasma MIP-1β levels in male PTSD patients vs male healthy controls. Error bars are ±s.e.m. *P*-values for the difference at each hour are shown in **d**. PTSD males are trend slightly greater than healthy control males at every hour. However, the differences at every hour are not significant across circadian time,(*P⩽*0.05). Note that healthy control males differ from healthy control females by being intrinsically elevated into levels of MIP-1β comparable to female PTSD patients. (**c**) Differences between average log plasma MIP-1β levels in all PTSD patients vs all healthy controls. Error bars are ±s.e.m. *P-*values for the difference at each hour are shown in **d**. All PTSD patients trend greater than all healthy controls at every hour. However, this trend is based on the contribution from female PTSD patients only. (**d**) Significance of log plasma concentration differences for MIP-1β over circadian time. Vertical axis is log *P-*value. Red horizontal line is the same as *P*=0.05 on a arithmetic scale. Male PTSD (dotted black line); female PTSD (dashed black line); all PTSD (solid black line). Only female PTSD differ significantly from female healthy controls at every hour across circadian time.

Figure 5 shows the complete circadian profile for MIP-1β in both PTSD and healthy control patients. As shown in Figure 5a, and in the statistical profile in Figure 5d, female PTSD patients have significantly elevated levels of plasma MIP-1β across the entire circadian interval. By contrast, as shown in Figure 5b, and the statistical profile in Figure 5d, male PTSD patients do not differ significantly from male healthy controls across the entire circadian interval. Furthermore, the data show that male MIP-1β plasma levels on both PTSD and healthy controls have average values in the vicinity of female PTSD plasma levels. Because of differences in numbers of females vs males in this study, the gender-independent analysis (Figure 5c) is heavily weighted by the female data. Thus plasma MIP-1β can only be considered as a candidate plasma marker for PTSD in females.

**Figure 6:**
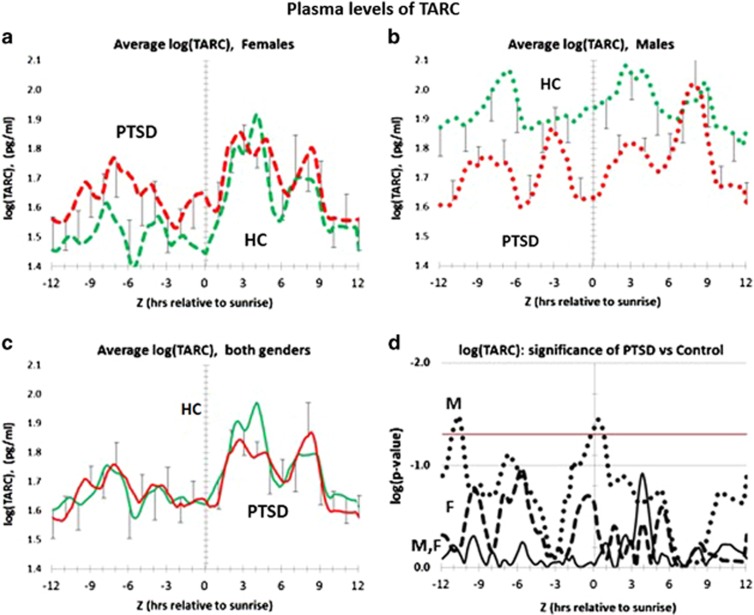
Distribution of plasma levels of TARC over a circadian interval for patients with PTSD and healthy controls. (**a**) Differences between average log plasma TARC levels in female PTSD patients *vs* female healthy controls. Error bars are ±s.e.m. *P-*values for the difference at each hour are shown in **d**. Differences between PTSD Females and healthy control females at every hour are not significant (*P*⩽0.05). (**b**) Differences between average log plasma TARC levels in male PTSD patients are lower than male healthy controls. Error bars are ±s.e.m. *P-*values for the difference at each hour are shown in **d**. PTSD males are differ significantly at each hour across circadian time from healthy controls only at ca. *Z*−12 and *Z*, (*P*⩽0.05). (**c**) Differences between average log plasma TARC levels in all PTSD patients vs all healthy controls. Error bars are ±s.e.m. *P-*values for the difference at each hour are shown in **d**. There are no significant differences at each hour across circadian time, (*P*⩽0.05). (**d**) Significance of log plasma concentration differences for TARC lover circadian time. Vertical axis is log *P-*value. Red horizontal line is the same as *P*=0.05 on a arithmetic scale. Male PTSD (dotted black line); female PTSD (dashed black line); all PTSD (solid black line). Male PTSD patients differ significantly from male healthy controls at every hour across circadian time only at ca. *Z* -12 and *Z*, (*P*⩽0.05). HC, healthy controls; PTSD, post-traumatic stress disorder.

Supplementary Figure 6 tests for MIP-1β entrainment differences between PTSD and healthy controls. The data show that ca. 80% of PTSD and healthy controls peak just after sunrise ((*Z*=3) h). Entrainment differences for females (Supplementary Figure 6a) and males (Supplementary Figure 6b) appear similar. In addition, Supplementary Figure 6d shows that these differences only trend towards significance, except for PTSD females at (*Z*=−3) hours. Because of differences in numbers of females vs males in this study, the gender-independent analysis (Figure 6c) is heavily weighted by the female data.

### Gender-dependent effect of PTSD on the circadian profiles for plasma TARC

Figure 6 shows the complete circadian profile for TARC in both PTSD and healthy control patients. As shown in Figure 6a, and in the statistical profile in Figure 6d, female PTSD patients have trending elevated levels of plasma TARC mostly before, but not after sunrise. By contrast, Figure 6b shows that male PTSD patients are systematically lower than male healthy controls. This male-specific difference becomes significant at (*Z*−12) hours, and at sunrise ((*Z*=0) h). PTSD males also have a novel peak at (*Z*=−3) h. The gender-independent differences become statistically insignificant because of the differences in direction of TARC levels in either gender (Figure 6c). Thus plasma TARC can only be considered as a candidate plasma marker for PTSD in males.

Supplementary Figure 7 tests for whether there are TARC entrainment differences between PTSD and healthy controls. Supplementary Figures 7a and 7b show that 40–60% of both male and female patients, independent of PTSD, have broad peaks centered at (*Z*−7) and (*Z*+5) h. For PTSD patients, independent of gender, 100% of the populations have peaks at (*Z*+3) h. However, as shown in Supplementary Figure 7d, significant differences between PTSD and healthy controls only occasionally reach significance. Finally, a mixture of genders essentially reflects contributions from female patients due to differences in patient numbers.

### Effect of PTSD on the circadian profiles for plasma IP-10, Eotaxin and IL-8

The complete circadian profiles for IP-10 (Supplementary Figure 8), Eotaxin (Supplementary Figure 9) and IL-8 (Supplementary Figure 10) do not indicate significant PTSD-specific differences in either male or female patients. In the case of IP-10, female PTSD patients are elevated relative to healthy controls (Supplementary Figure 10a), while males are the reverse. However, *P-*values (Supplementary Figure 10d) show that female PTSD patients only trend towards a significant difference. In addition, approximately 100% of all patients show an entrainment maximum for IP-10 in the vicinity of sunrise ((*Z*=0)). By contrast, there is virtually no difference between PTSD and healthy controls for Eotaxin. Furthermore, the entrainment analysis for IP-10 indicates that virtually all patients have a minimum at sunrise (*Z*=0). The value of these data for IP-10 and Eotaxin include not only the fact that they are unique in the literature, but that their entrainment amplitudes are exactly 12 h out of phase with each other.

In the case of IL-8, levels of IL-8 trend towards elevation in PTSD, but never reach significance. A second feature of the IL-8 distribution is that levels of IL-8 are higher in males (an average across circadian time of ca. 3.5 pg/ml) than in females (an average across circadian time of ca. 2.5 pg ml^−1^). In IL-8 entrainment studies, ca. 70% of both males and females show a broad peak in the region of (2<*Z*<9) hours, with evidence of a second peak at (9<*Z*<12) hours. Consistently, this double post-sun-up peak has been documented for IL-8 in the past.^26^

### Measurement of cytokines and chemokines in PTSD CSF at 0900 hours

Supplementary Table 3 shows measurements of cytokines and chemokines in CSF, which were collected from both PTSD and healthy control patients at the 0900 hours time point. Of the complete set of analytes, only IL-8 was able to come close to being significantly reduced in PTSD CSF. While IL-8 in PTSD CSF was reduced by ca. 25%, the *P-*value (two-tailed) was 0.06. Surprisingly, we find that in the 0900 hours CSF samples, the MCP-4 and MCP-1 levels are the reverse of those found in plasma, and independent of PTSD. Specifically, in healthy control CSF, the MCP-1 levels are ca. 7-fold higher than in plasma, while the MCP-4 levels are ca. 100-fold lower. Furthermore, independently of the quantitative levels, the MCP-4/MCP-1 ratio in CSF does not significantly discriminate between PTSD and healthy control patients, as it does in the 0200 or 0900 hours plasma samples. Thus while the MCP-4/MCP-1 ratio appears to be a very good candidate biomarker for PTSD in plasma, it fails in CSF. Thus, the respective mechanisms responsible for setting CSF and plasma levels of MCP-1 and MCP-4 in PTSD and healthy controls appear to be independent of each other.

### Mixed model statistical analysis of PTSD *vs* healthy controls

Supplementary Figure 11 and Supplementary Table 6 show the consequences of using a mixed model statistical analysis, in which the data are further simplified by reducing the number of daily time points from 24 to just 6. These are morning (3 h after sunrise), noon (7 h after sunrise), afternoon (11 h after sunrise), evening (15 h after sunrise), and early morning (23 h after sunrise). For each one of these time points (‘zeits') we used linear regression of the data points in the span of 2 h before to 2 h after the given time. These 6 interpolated data points for each study subject were then used as input to the Mixed Model analysis (Supplementary Table 6). The results of the mixed model analysis show that after adjusting for multiple repeats, using the most restrictive Bonferroni adjustments of the PTSD group *vs* controls, the difference in the MCP-4/MCP-1 ratio between PTSD and healthy controls remains highly significant (*P*=0.026; Supplementary Figure 11a.). Consistently, based on only six averaged time points the time of day remains a significant determinant of the MCP-4/MCP-1 ratio (*P*=0.0003; Supplementary Figure 11a), where the difference is seen by comparing the similarity of of the peak and the trough times for the PTSD and healthy controls.

By contrast, the age at the time of trauma, which we define as pre-pubertal *vs* post-pubertal (discrimination age=11YO), does not seem to have a significant effect on the MCP-4.MCP-1 ratio in the PTSD group (*P*= 0.18, see Supplementary Figure 11b). In addition. there is no significant difference between the PTSD patients and healthy controls in terms of circadian behavior (*P*=0.53, see Supplementary Figure 11c). With respect to Supplementary Figure 11c, although the means seem to suggest differences, the variances indicate that we are still insufficiently powered to significantly discriminate between the three categories. Furthermore, the analysis indicate that gender does not have a significant effect on the MCP-4/MCP-1 ratio (*P*=0.27). Finally, BMI (discriminators: normal<25 kg/m^2^; overweight 25–30 kg/m^2^; obese>30 kg/m^2^) has no significant effect on the temporal behavior of the MCP-4/MCP-1 ratio either as a continuous variable (*P*=0.66) or as a categorical variable (*P*=0.36). This alternative mixed model approach to the analysis validates the analysis of variance approach used in other parts of this paper, and adds specific information on the lack of significant influence of the age of trauma on the circadian behavior of the PTSD-dependent MCP-4/MCP-1 ratio.

## Discussion

We conclude, based on both analysis of variance and mixed model statistical analyses, that the MCP-4/MCP-1 ratio in plasma is a significant, quantitative discriminator between PTSD patients and healthy controls. This effect is not significantly dependent on circadian time of day, gender, BMI or age in which the trauma is suffered. By contrast, the monocyte chemokine MIP-1β significantly distinguishes only between female PTSD patients and female healthy controls across the entire 27 h sampling period. However, in male PTSD patients, TARC, a lymphocyte chemokine released from monocytes, is significantly reduced only at *Z* – 6 h (about midnight) and sunrise time (*Z*=0). There is also trending evidence that the circadian rhythms for the MCP-1/MCP-4 ratio, MIP-1β and TARC may be shifted forward in time. These trends can be seen in entrainment plots of all three analytes for significant differences between PTSD and healthy controls in *Z*=ca. 9−12 h, circadian time (viz., late afternoon to evening, local time). Importantly, plasma levels of three other chemokines (IP-10, Eotaxin, and IL-8) are not significantly affected by PTSD at any time, thus suggesting some specificity for those monocyte chemokines that distinguish PTSD patients from healthy controls.

### PTSD effects on variation in plasma MCP-4, MCP-1 and the MCP-4/MCP-1 ratio

It has been shown in animal models that an acute severe stressor can induce increased concentrations of inflammatory cytokines and chemokines, including MCP-1.^27^ Both MCP-4 (CCL13) and MCP-1 (CCL2) share 67% sequence homology, and function as molecular attractants (‘chemokines') for monocytes, and to a lesser extent for lymphocytes and basophils. Both chemokines also share CCR2 as a common receptor.^28, 29^ Therefore, the fact that the MCP-4/MCP-1 ratio, at any time, biomarks the plasma from PTSD patients can direct our attention to the existence of precedents for possible defects in monocytes and other immune cells associated with PTSD. Segman *et al.*^14^ studied peripheral blood mononuclear cells in survivors of terror attacks in Israel. Yehuda *et al.*^15^ studied whole blood expression patterns in survivors of the World Trade Center attack in New York. Neylan *et al.*^13^ studied purified CD14^+^ monocytes from men and women with PTSD and other comorbidities. In all three cases, a common observation was suppression of gene expression. The timing of blood collection is not mentioned in these publications, although morning collections might have been expected. Nonetheless, it remains a challenge to understand exactly how PTSD symptoms might correlate with dysfunctional gene expression in immune cells.

However, recent studies indicate that circulating monocytes exhibit a circadian oscillation, coinciding with endogenous MCP-1 expression. The oscillation is driven by an autonomous circadian clock, for which the time-dependent variation is independent of infection or metabolic stress.^24, 25^ In humans, nocturnal peak blood levels, encompassing the time period between 0100 and 0300 hours are observed for circulating monocytes, and for both T and B lymphocytes.^12^ Transcriptionally, nocturnal monocytes activate the expression of MCP-1.^24^ Beginning at ca. 0400 hours, levels of circulating monocytes, and both B and T cells, begin to decline.^12^ Coincidently, monocyte expression of MCP-1 is blocked by the transcription factors CLOCK, BMAL1 and EZH2.^24^ As predicted from this mechanism, MCP-1 plasma levels of healthy controls significantly drop from 0200 to 0900 hours by ca.70% (*P*=0.001) (Supplementary Table 5 and Figure 1). In the case of PTSD patients, MCP-1 levels also drop, but by a greater proportion (ca. 90%), and with greater significance (*P*=2 × 10^−^^6^). This process is also seen in greater detail in the circadian pattern (Figure 3c). It is therefore possible that this disease-specific difference is the dynamic basis for MCP-1 contributing to the lower denominator portion of the PTSD-specific MCP-4/MCP-1 biomarker.

Less is known about the genetics, or possible circadian variation of plasma MCP-4. However, as shown in Supplementary Table 4, our data show that MCP-4 plasma levels also significantly decrease from 0200 to 0900 hours, local time. This decrease is by ca. 60% in healthy controls (*P*=0.004), compared with a significant decrease of only ca. 40% in PTSD patients (*P*=0.01). This process is also seen in greater detail in the *Z*-corrected circadian pattern (Figure 2d). Thus, the PTSD patients appear to express relatively reduced amounts of both MCP-1 and MCP-4 as the wake period begins, with a greater reduction in MCP-1 than for MCP-4. Thus, the elevation of the MCP-4/MCP-1 ratio in PTSD patients may be due to PTSD-dependent modifications in expression rates of both of these analytes.

### The disordered circadian rhythms for plasma MCP-1 and MCP-4 may identify a unique effect of PTSD

As emphasized earlier, sleep disturbance is considered to be a hallmark feature of PTSD.^3, 4, 5^ It is therefore possible that sleep deprivation, *per se*, might be the cause of the disordered circadian profile for MCP-4 and MCP-1. However, in a comprehensive study of ten fully instrumented normal males, Born *et al.*^12^ reported that following a 24 h sleep deprivation experience, the succeeding 24 h were characterized only by a blunting of the extents of circadian changes in immune cell numbers, including monocytes, and in TNFα, IL-1β, and IL-6 levels, but not by changes in phase or appearance of multimodality. Thus, the circadianopathy observed here for PTSD patients for plasma MCP-4 and MCP-1 would appear to be disease-related. Furthermore, the fact that there are no observed PTSD-specific variations at the one available time point in any of the cytokines and chemokines in the CSF, may argue for a PTSD contribution to specific deficits in monocyte or immune cell biology. This conclusion is consistent with results mentioned above for peripheral blood mononuclear cell^14^ and whole blood,^15^ respectively, and for CD14^+^ mononuclear cells.^13^ However, this possibility still does not explain how the central PTSD condition might translate into a peripheral circadian dysfunction.

The central clock mechanism in the brain is run by light exposure and activation of CLOCK/BMAL1 signaling in the suprachiasmatic nucleus.^30^ Subsidiary clocks in the periphery take their cue from this central mechanism, via the hypothalamic–pituitary–adrenal axis, and adapt the exact subsidiary timing to their own requirements. In healthy controls, the circadian clock program is intrinsically plastic, and changes, such as those induced by changes in metabolism, or sunrise time, are reversible.^31^ By contrast, chronic neuropsychiatric disorders have been associated with conditions in which the central clock appears to permanently ‘lose track of time'.^32^ Therefore, based on present data, the mechanisms linking central PTSD to individual peripheral circadian clock dysfunctions for MCP-1 or MCP-4 remain to be discovered.

However, we need to emphasize here that there are limitations to this study. Not the least among these are a limited sample size and unequal numbers of male and female participants. In fact, we were unable to significantly discriminate between male and female subjects for any of the 17 individual analytes at either 0900 or 0200 hours, local time, in healthy controls or PTSD patients. The differences we do find, as for MIP-1β and TARC, have significance that only intermittently exceed the minimum *P*=0.05 level, and certainly not the Bonferroni correction criterion. Therefore, if there are generalized gender-specific differences in the circadian distribution for these analytes, we are underpowered to identify them. As a final caveat, it should be emphasized that this is a pioneer study, and that larger numbers of healthy control and PTSD patients need to be studied in this longitudinal manner to validate the conclusions. On a positive note, this patient cohort was selected on the basis of low comorbidity for MDD, and at least a six months separation from alcohol or drug use.^1^ By contrast, many previous studies have been forced to utilize heterogeneous patient cohorts, with MDD and other comorbidities objectively present. On a further positive note, the strategy of hourly sample collections over 24 h has allowed us to collect high-resolution data, which reveal significant similarities among the healthy controls and PTSD patients, at specific times, as well significant quantitative and qualitative differences between them.

### Cytokines and chemokines in CSF from PTSD and healthy control patients

By contrast, data presented here show that few PTSD-specific changes in CSF can be detected from simply screening cytokines and chemokines at 0900 hours. In fact, the only difference we detected was a small, trending reduction in IL-8. These data are therefore consistent with data reported by Bonne *et al.*^1^ for a subset of cytokines in the same CSF samples, The caveat here is that CSF from other times were not collected. We therefore cannot exclude the possibility that cytokine or chemokine signals in CSF may exist in PTSD.

In the meantime, as summarized in Supplementary Table 6, we have documented remarkable concentration gradients between 0900 hours CSF and plasma. The most profoundly elevated gradients are for IL-8 (elevated ca. 14-fold); for MCP-1 (elevated ca. 7-fold); for IP-10 (elevated ca. 7-fold); for MIP-1β and for Eotaxin 3 (both elevated ca. 4-fold). The most profoundly reduced analytes were MCP-4 (reduced ca. 80–100-fold); Eotaxin 1 (reduced ca. 18-fold); MDC (reduced ca. 25-fold); TARC (reduced ca. 9-fold); and TNFα (reduced ca. 6-fold). As mentioned earlier, we do not yet understand how these gradients are generated or sustained. These gradients have not previously been reported either in PTSD or healthy control subjects.

## Conclusions

It has been hypothesized that proinflammatory cytokines and chemokines in the CSF or plasma might be biomarkers, surrogates, or drivers for the psychophysiological mechanisms relating a history of trauma exposure to changes in behavior and mental health disorders, and medical morbidity. However, in this relatively comprehensive test, significant changes can be detected in plasma, but not CSF. By carefully correcting all data for the time of sunrise, small but significant changes in expression level and entrainment can be detected for the MCP-4/MCP-1 ratio (both genders), MIP-1β (females) and TARC (males), but not other tested chemokines. Thus a central effect, consistent with previously published reciprocal changes in plasma cortisol and ACTH may be inferred.^7^ Prospectively, the mechanistic origins of this dysfunction, and their relationship to PTSD, remain here for future study.

## Figures and Tables

**Table 1:** Cytokine and chemokine concentrations in plasma samples from PTSD and healthy controls at 0900 hours (pg ml^−1^)

*Analyte*	*PTSD*^a^	*HC*^a^	P*-value*^b^	*Ratio*^c^	*AUC*^d^
GM-CSF	< LLOD	< LLOD	NA	NA	NA
IFN-γ	< LLOD	< LLOD	NA	NA	NA
IL-10	0.79±0.24	0.69±0.32	0.21	1.15±0.60	0.69
IL-12 p70	< LLOD	< LLOD	NA	NA	NA
IL-1β	0.08±0.02	0.18±0.04	0.04	↓ 2.12±0.63	0.71
IL-2	0.15±0.04	0.17±0.05	0.72	0.93±0.38	0.55
IL-6	0.47±0.04	0.77±0.16	0.15	↓ 1.62±0.33	0.74
IL-8	1.67±0.56	1.98±0.59	0.67	0.84±0.37	0.52
TNF-α	2.57±0.41	1.57±0.22	0.03	↑ 1.64±0.34	0.76
Eotaxin	503.45±37.95	496.61±37.29	0.91	1.01±0.11	0.52
Eotaxin-3	6.90±0.55	6.42±0.38	0.58	1.07±0.10	0.63
IP-10	226.06±30.53	149.66±13.37	0.04	↑ 1.51±0.24	0.73
MCP-1	171.11±17.01	207.10±17.49	0.14	0.83±0.11	0.82
MCP-4	298.35±27.00	208.62±23.94	0.01	↑ 1.43±0.20	0.77
MDC	1850.02±155.12	1680.60±167.21	0.40	1.10±0.14	0.61
MIP-1β	65.85±8.12	55.58±4.77	0.36	1.18±0.17	0.66
TARC	85.52±13.86	67.32±10.24	0.30	↑ 1.27±0.28	0.70
MCP1/MCP4	0.61±0.06	1.12±0.18	4E−03	↓ 1.84±0.33	0.84
MCP4/MCP1	1.82±0.19	1.10±0.16	4E−03	↑ 1.66±0.28	0.84

Abbreviations: CSF, cerebrospinal fluid; HC, healthy controls; IFN-γ, interferon γ IL, interleukin; LLOD, lower limit of detection; NA, not applcable; PTSD, post-traumatic stress disorder; ROC, receiver operation condition; TNF-α, tumor necrosis factor α.

a Average±s.e.m., pg ml^−1^.

b Two-tailed *t*-test.

c ↑PTSD>HC;↓PTSD<HC.

d Area under the curve (AUC) of the ROC Curve.
