# Supplementary material for: The MCP-4/MCP-1 ratio in plasma is a candidate circadian biomarker for chronic post-traumatic stress disorder
Source: Transl Psychiatry. 2017 Feb 7;7(2):e1025–. doi: 10.1038/tp.2016.285 (PMC5438024; doi:10.1038/tp.2016.285)
Supplement: Supplementary Figure Legends [file tp2016285x1.docx]

**Legends for Supplementary Figures**

**The MCP-4/MCP-1 Ratio in Plasma is a Candidate Circadian Biomarker for** **Chronic Post-Traumatic Stress Disorder**

Clifton Dalgard ^1,2, &^ , Ofer Eidelman ^1,2,&^, Catherine Jozwik ^1,2^, Cara H. Olsen ^4^, Meera Srivastava ^1,2^, Roopa Biswas ^1,2^ , Yvonne Eudy ^1,2^, Stephen W. Rothwell ^1,2^, Gregory P. Mueller ^1,2^, Peixiong Yuan ^3^, Wayne Drevets ^3,#^, Husseini K. Manji^3,#^, Meena Vythlingam ^3,@^, Dennis S. Charney ^3,*^ , Robert J. Ursano ^4,5^, David M. Jacobowitz ^1,2^, Harvey B. Pollard ^1,2,%^  , and Omer Bonne ^6,%^ .

**Supplementary Figure 1**. **Time Courses for plasma cortisol and ACTH in a separate cohort of PTSD and Healthy Control patients (data from Vythilingam et al, 2010 [**[**1**](#_ENREF_1)**]).**

**a**. Time course for plasma cortisol between 6:00PM amd 7:00 AM the next morning. Cortisol is consistently lower in PTSD patients than Healthy Controls over virtually the entire time course. Rise time is *ca*. 2 hours later than Healthy Controls

**b.** Time course for plasma ACTH between 6:00PM amd 7:00 AM the next morning. ACTH is consistently higher in PTSD patients than Healthy Controls over virtually the entire time course.

**c**. Enumeration of differences in time courses for cortisol and ACTH between 6:00PM amd 7:00 AM the next morning. These data are calculated by ourselves from the published Vythilingam et al (2010) [[1](#_ENREF_1)] time courses. The start of the ACTH increment is ca. 2 hours later than in Healthy Controls.

Reference:  Vythilingam M, Gill JM, Luckenbaugh DA, Gold PW, Collin C, et al. (2010) Low early morning plasma cortisol in posttraumatic stress disorder is associated with co-morbid depression but not with enhanced glucocorticoid feedback inhibition. Psychoneuroendocrinology 35: 442-450.

**Supplementary Figure 2**. **Discovery Series for plasma MCP-4/MCP-1 ratio over circadian time, base on 5 randomly selected PTSD patients and 5 randomly selected Healthy Controls**

**a**. Plasma MCP-4/MCP-1 ratio in discovery set of 5 PTSD and 5 Healthy Controls. Red solid line are average values for PTSD patients. Blue solid line are average values for Healthy Controls. Peripheral dotted lines are ± SEM ( standard error of the mean ).

**b**. Statistical significance in discovery set of 5 PTSD patients and 5 Healthy Controls of differences between selected plasma chemokines and MCP-4/MCP-1 ratio over circadian time. Color coded chemokines with p-values > 0.05 include IL-8, eotaxin, eotaxin-3, IP-10, MCP-1, MDC, and MIP-1β. The Chemokine MCP-4 (light blue) and the ratio of MCP-4/MCP-1 have P values < 0.01 across circadian time, Z.

**Supplementary Figure 3**. **Entrainment differences for MCP-4/MCP-1 ratio** **over a circadian interval for patients with PTSD and Healthy Controls**.

**a**. Percent of female PTSD patient s and female healthy Controls that are above their own daily average for plasma MCP-4/MCP-1 ratio. Red color is PTSD; green color is Healthy Control. Major peaks coincide at *ca*. 60% (at *ca*. Z-8 hours) and > 60% (at *ca*. Z + 3 hours). Differences between values at any hour. across circadian time ,are not significant (see **Supplementary** **Figure 3d**).

**b.** Percent of male PTSD patients and male healthy Controls that are above their own daily average for plasma MCP-4/MCP-1 ratio. Red color is PTSD; green color is Healthy Control. Differences between values at any hour. across circadian time ,are significant only at Z = +9 to +12. (see **Supplementary** **Figure 3d**).

**c**. Percent of all PTSD patients and all healthy Controls that are above their own daily average for plasma MCP-4/MCP-1 ratio. Red color is PTSD; green color is Healthy Control. Differences between values at any hour. across circadian time trend towards significance only at Z = +9 to +12. (see **Supplementary** **Figure 3d**).

**d**. Significance of differences between percent of PTSD patients and healthy Controls that are above their own daily average for plasma MCP-4/MCP-1 ratios. Vertical axis is log P value. Red horizontal line is the same as P = 0.05 on a arithmetic scale. Male PTSD (dotted black line); female PTSD (dashed black line); all PTSD (solid black line).

**Supplementary Figure 4**. **Entrainment differences for MCP-1 over a circadian interval for patients with PTSD and Healthy Controls**.

**a**. Percent of female PTSD patients and female healthy Controls that are above their own daily average for plasma levels of MCP-1. Red color is PTSD; green color is Healthy Control. Very few female PTSD patients have MCP-1 levels greater than their own daily averages in the time interval *ca*. [ Z -12], while ca. 70% of Female healthy Controls are elevated in this time interval. Both ends of the circadian profile. [Z= -12] and [Z = +12] agree. The significance for females trends towards significance (see **Supplementary Figure 4d**).

**b**. Percent of male PTSD patients and male healthy Controls that are above their own daily average for plasma levels of MCP-1. Red color is PTSD; green color is Healthy Control. Male PTSD patients have a 100% occupied peak at Z = -6 hours, compared to only 20% of Healthy Control males at this time region. The maximum significant difference is in the preceding time interval of [-12 < Z < -9] hours (see **Supplementary Figure 4d**). The percent is pushed forward by ca. 2 hours in the time region of [+6 < Z < +9] hours, with a trend towards significance for the difference in this time interval

**c**. Percent of all PTSD patients and all healthy Controls that are above their own daily average for plasma levels of MCP-1. Red color is PTSD; green color is Healthy Control. As group, the peaks for at least 80% of both patients and healthy controls is a -8 < Z < 0) hours, with a progressive reduction following sunrise [Z = 0].

**d**. Significance of differences between percent of PTSD patients and healthy Controls that are above their own daily average for plasma levels of MCP-1. Vertical axis is log P value. Red horizontal line is the same as P = 0.05 on a arithmetic scale. Male PTSD (dotted black line); female PTSD (dashed black line); all PTSD (solid black line). Differences are significant for males in the region around Z = 12 hours.

**Supplementary Figure 5**. **Entrainment differences for MCP-4 over a circadian interval for patients with PTSD and Healthy Controls**.

**a**. Percent of female PTSD patients and female healthy Controls that are above their own daily average for plasma levels of MCP-4. Red color is PTSD; green color is Healthy Control. Major peaks coincide at *ca*. 60% (at *ca*. Z-8 hours) and > 80% (at *ca*. Z + 3 hours). Differences across circadian time are not significant (see **Supplementary Figure 5d**).

**b.** Percent of male PTSD patients and male healthy Controls that are above their own daily average for plasma levels of MCP-4. Red color is PTSD; green color is Healthy Control. At least 80% (4 of 5 males) are above their own means, and above those of male healthy controls [+8 < Z < +12] hours. The difference is significant (see **Supplementary Figure 5d**).

**c**. Percent of all PTSD patients and all healthy Controls that are above their own daily average for plasma levels of MCP-4. Red color is PTSD; green color is Healthy Control. The gender-independent differences between PTSD and Healthy control can be seen in the time region [+8 < Z < +12] hours, and trend towards significance (see **Supplementary Figure 5d**, solid line marked **M,F**).

**d**. Significance of differences between percent of PTSD patients and healthy Controls that are above their own daily average for plasma levels of MCP-4. Vertical axis is log P value. Red horizontal line is the marks P = 0.05 on a logarithmic scale. Male PTSD (dotted black line); female PTSD (dashed black line); all PTSD (solid black line). Differences are significant for male PTSD patients, and trend towards significance for all PTSD patients in the time domain [+8 < Z < +12].

**Supplementary Figure 6**. **Entrainment differences for MIP-1β over a circadian interval for patients with PTSD and Healthy Controls**.

**a**. Percent of female PTSD patient s and female healthy Controls that are above their own daily average for plasma levels of MIP-1β. Red color is PTSD; green color is Healthy Control. The peak values for 80% of the female, both PTSD and Healthy Controls, is in the time zone of [-3 < Z >+6] hours. The center of gravity of this distribution seems to be shifted towards later times by *ca*. 2 hours. However, no differences at each hour, over circadian time, approach significance (p < 0.05) (see **Supplementary Figure 6d**).

**b.** Percent of male PTSD patients and male healthy Controls that are above their own daily average for plasma levels of MIP-1β. Red color is PTSD; green color is Healthy Control. Both PTSD and Healthy Control males peak at *ca* 80% in the time zone [0 < Z < +6] hours. Differences between male PTSD and male Healthy Controls are significant only at [+6 < Z < +9] hours ((see **Supplementary Figure 6d**). As for the female PTSD patients, the distribution for male PTSD patients appear to shift to later time by *ca*. 2 hours.

**c**. Percent of all PTSD patients and all healthy Controls that are above their own daily average for plasma levels of MIP-1β. Red color is PTSD; green color is Healthy Control. Both PTSD and Healthy Control patients peak at 100% in the time zone [-3 < Z < +3] hours. Differences at any hour over circadian time between all PTSD and all Healthy Controls are significant only at [Z -3] and [Z +10] hours (see **Supplementary Figure 6d**). As for both male and female PTSD patients, the distribution appears to shift to later time by *ca*. 2 hours.

**d**. Significance of differences between percent of all PTSD patients and all Healthy Controls that are above their own daily average for plasma levels of MIP-1β. Vertical axis is log P value. Red horizontal line is the same as P = 0.05 on a arithmetic scale. Male PTSD (dotted black line); female PTSD (dashed black line); all PTSD (solid black line).

**Supplementary Figure 7**. **Entrainment differences for TARC over a circadian interval for patients with PTSD and Healthy Controls**.

**a**. Percent of female PTSD patients and female healthy Controls that are above their own daily average for plasma levels of TARC l. Red color is PTSD; green color is Healthy Control. A maximum for female Healthy Controls are found at [-9 < Z < -3] hours, and a bifid maximum in the time zone of [+3 < Z < +9] hours. For female PTSD, the nighttime maximum is also found, but significantly differs from the PTSD signal at Z -6 hours (p < 0.05) (see **Supplementary Figure 7d**). However, while the PTSD bifid daytime maxima are blunted, the hourly differences are not significant (p < 0.05).

**b.** Percent of male PTSD patients and male healthy Controls that are above their own daily average for plasma levels of TARC l. Red color is PTSD; green color is Healthy Control. Male PTSD patients and Healthy Controls distributed into a nighttime and a daytime maximum. Significant differences between male PTSD and male Healthy Controls were found for Z -12, Z-3, and Z hours (see **Supplementary Figure 7d**). .

**c**. Percent of all PTSD patients and all healthy Controls that are above their own daily average for plasma levels of TARC. Red color is PTSD; green color is Healthy Control. Patterns of all PTSD and all Healthy Controls substantially resemble that for female PTSDS and Healthy Controls. No significant differences were noted (p < 0.05) (see **Supplementary Figure 7d**). .

**d**. Significance of differences between percent of PTSD patients and healthy Controls that are above their own daily average for plasma levels of TARC l. Vertical axis is log P value. Red horizontal line is the same as P = 0.05 on a arithmetic scale. Male PTSD (dotted black line); female PTSD (dashed black line); all PTSD (solid black line).

**Supplementary Figure 8**. **Distribution and** **entrainment differences for IP-10 over a circadian interval for patients with PTSD and Healthy Controls**.

**a**. Differences between average log plasma IP-10 levels in female PTSD patients vs female healthy controls. Error bars are ± SEM (standard error of the mean). P values for the difference at each hour are shown in **Figure 1d**. Female PTSD patients are systematically higher than female healthy controls. However, the differences only trend towards significance (p < 0.05).

**b**. Differences between average log plasma IP-10 levels in male PTSD patients vs male healthy controls. Error bars are ± SEM (standard error of the mean). P values for the difference at each hour are shown in **Figure 1d**. Male PTSD patients are systematically lower than male healthy controls. However, the hourly differences are not significant (p < 0.05).

**c**. Differences between average log plasma IP-10 levels in all PTSD patients vs all healthy controls. Error bars are ± SEM (standard error of the mean). P values for the difference at each hour are shown in **Figure 1d**. All PTSD patients are higher than all Healthy Controls. However, the hourly differences are not significant (p < 0.05).

**d**. Significance of log plasma concentration differences for IP-10 over circadian time. Vertical axis is log P value. Red horizontal line is the same as P = 0.05 on a logarithmetic scale. Male PTSD (dotted black line); female PTSD (dashed black line); all PTSD (solid black line).

**e**. Percent of female PTSD patient s and female Healthy Controls that are above their own daily average for plasma levels of MCP-4. Red color is PTSD; green color is Healthy Control. Approximately 90% of both female PTSD patients and female Healthy Controls have a peak at Z hours. Hourly differences were not significant (p < 0.05).

**f.** Percent of male PTSD patients and male Healthy Controls that are above their own daily average for plasma levels of IP-10. Red color is PTSD; green color is Healthy Control. Approximately 100% of both male PTSD patients and male Healthy Controls have a peak in the vicinity of Z hours. Hourly differences were not significant, except for a signal at Z – 8 hours. (p < 0.05). However the significant difference was evident for only a small fraction of the male PTSD patients.

**g**. Percent of all PTSD patients and all Healthy Controls that are above their own daily average for plasma levels of IP-10. Red color is PTSD; green color is Healthy Control. Approximately 100% of all PTSD patients and all Healthy Controls have a peak in the vicinity of Z hours. Hourly differences were not significant (p < 0.05).

**h**. Significance of differences between percent of PTSD patients and Healthy Controls that are above their own daily average for plasma levels of IP-10. Vertical axis is log P value. Red horizontal line is the same as P = 0.05 on a arithmetic scale. Male PTSD (dotted black line); female PTSD (dashed black line); all PTSD (solid black line).

**Supplementary Figure 9**. **Distribution and** **entrainment differences for Eotaxin over a circadian interval for patients with PTSD and Healthy Controls**.

**a**. Differences between average log plasma Eotaxin levels in female PTSD patients vs female healthy controls. Error bars are ± SEM (standard error of the mean). P values for the difference at each hour are shown in **Figure 9d**. Hourly differences between female PTSD and female Healthy controls are not significant (p < 0.05).

**b**. Differences between average log plasma Eotaxin levels in male PTSD patients vs male Healthy controls. Error bars are ± SEM (standard error of the mean). P values for the difference at each hour are shown in **Figure 9d**. Hourly differences between male PTSD and male Healthy controls are not significant (p < 0.05).

**c**. Differences between average log plasma Eotaxin levels in all PTSD patients vs all healthy controls. Error bars are ± SEM (standard error of the mean). P values for the difference at each hour are shown in **Figure 9d**. Houtly differences between all PTSD and all Healthy controls are not significant (p < 0.05).

**d**. Significance of log plasma concentration differences for Eotaxin over circadian time. Vertical axis is log P value. Red horizontal line is the same as P = 0.05 on a arithmetic scale. Male PTSD (dotted black line); female PTSD (dashed black line); all PTSD (solid black line).

**e**. Percent of female PTSD patients and female Healthy Controls that are above their own daily average for plasma levels of Eotaxin. Red color is PTSD; green color is Healthy Control. Nearly 100% of the female PTSD and Healthy Controls are maximal at Z < -9 hours and Z + 9 hours. A minimum for both female PTSD and Healthy Controls is seen at Z hours. No significant hourly differences are evident (p < 0.05).

**f**. Percent of male PTSD patients and male Healthy Controls that are above their own daily average for plasma levels of Eotaxin. Red color is PTSD; green color is Healthy Control. A pattern very similar to that for female PTSD patients is evident. No significant hourly differences are evident. (p < 0.05).

**g**. Percent of all PTSD patients and all healthy Controls that are above their own daily average for plasma levels of Eotaxin. Red color is PTSD; green color is Healthy Control. The distribution parallels that for female PTSD *vs* female Healthy Controls (see **Figure 3Se**).

**h**. Significance of differences between percent of PTSD patients and healthy Controls that are above their own daily average for plasma levels of Eotaxin. Vertical axis is log P value. Red horizontal line is the same as P = 0.05 on a arithmetic scale. Male PTSD (dotted black line); female PTSD (dashed black line); all PTSD (solid black line).

**Supplementary Figure 10**. **Distribution and** **entrainment differences for IL-8 over a circadian interval for patients with PTSD and Healthy Controls**.

**a**. Differences between average log plasma IL-8 levels in female PTSD patients vs female healthy controls. Error bars are ± SEM (standard error of the mean). P values for the difference at each hour are shown in **Figure 10d**. Hourly differences between female PTSD and female Healthy controls are not significant (p < 0.05).

**b**. Differences between average log plasma IL-8 levels in male PTSD patients vs male Healthy controls. Error bars are ± SEM (standard error of the mean). P values for the difference at each hour are shown in **S** **Figure 10d**. Hourly differences between male PTSD and male Healthy controls are not significant (p < 0.05). However, levels of plasma IL-8 in males are generally *ca*. 25% higher than in females.

**c**. Differences between average log plasma IL-8 levels in all PTSD patients vs all healthy controls. Error bars are ± SEM (standard error of the mean). P values for the difference at each hour are shown in **S** **Figure 10d**. Hourly differences between all PTSD and all Healthy controls are not significant (p < 0.05). What can be said is that there is slight trend towards higher IL-8 in PTSD patients, independent of gender.

**d**. Significance of log plasma concentration differences for IL-8 over circadian time. Vertical axis is log P value. Red horizontal line is the same as P = 0.05 on a arithmetic scale. Male PTSD (dotted black line); female PTSD (dashed black line); all PTSD (solid black line).

**e**. Percent of female PTSD patients and female Healthy Controls that are above their own daily average for plasma levels of IL-8. Red color is PTSD; green color is Healthy Control. Approximately 80% of the female PTSD and Healthy Controls are maximal at [3 < Z < 9 hours]. A minimum for both female PTSD and Healthy Controls is seen at [-3 > Z > -9 hours]. No significant hourly differences are evident (p < 0.05). Afternoon peaks in IL-8 are consistent with previous literature.

**f**. Percent of male PTSD patients and male Healthy Controls that are above their own daily average for plasma levels of IL-8. Red color is PTSD; green color is Healthy Control. A very “noisy” pattern is seen compared with female PTSD. Some significant hourly differences are evident at [Z = -4 hours] and [Z = -7 hours] (p < 0.05) for 80% of the males.

**g**. Percent of all PTSD patients and all healthy Controls that are above their own daily average for plasma levels of IL-8. Red color is PTSD; green color is Healthy Control. The distribution parallels that for female PTSD *vs* female Healthy Controls (see **S** **Figure 10e**).

**h**. Significance of differences between percent of PTSD patients and healthy Controls that are above their own daily average for plasma levels of IL-8. Vertical axis is log P value. Red horizontal line is the same as P = 0.05 on a arithmetic scale. Male PTSD (dotted black line); female PTSD (dashed black line); all PTSD (solid black line). Caution regarding statistics for males IL-8 balues is needed because of fewer males than female patients.

**Supplementary Figure 11. Circadian behavior of the average MCP-4/MCP-1 data points used in the mixed model analysis.**

**a.** Circadian changes in MCP-4/MCP-1 ratio.  The vertical shift between the PTSD and the control groups is very significant (p = 0.0003)**.**

**b.** Analysis of age of PTSD patient at time of trauma. Discrimination is on the basis of pre-pubertal vs post-pubertal trauma age.  Difference is not significant **(**p=0.18).

**c.** The temporal behavior of the MCP-4/MCP-1 ratio, apart from the vertical shift, is not significantly different between the various groups of patients and the controls (p=0.53).  The curves from **Panel b** were normalized by subtracting the respective daily average from each data point.
