# Supplementary figures and images for: The MCP-4/MCP-1 ratio in plasma is a candidate circadian biomarker for chronic post-traumatic stress disorder
Source: Transl Psychiatry. 2017 Feb 7;7(2):e1025–. doi: 10.1038/tp.2016.285 (PMC5438024; doi:10.1038/tp.2016.285)

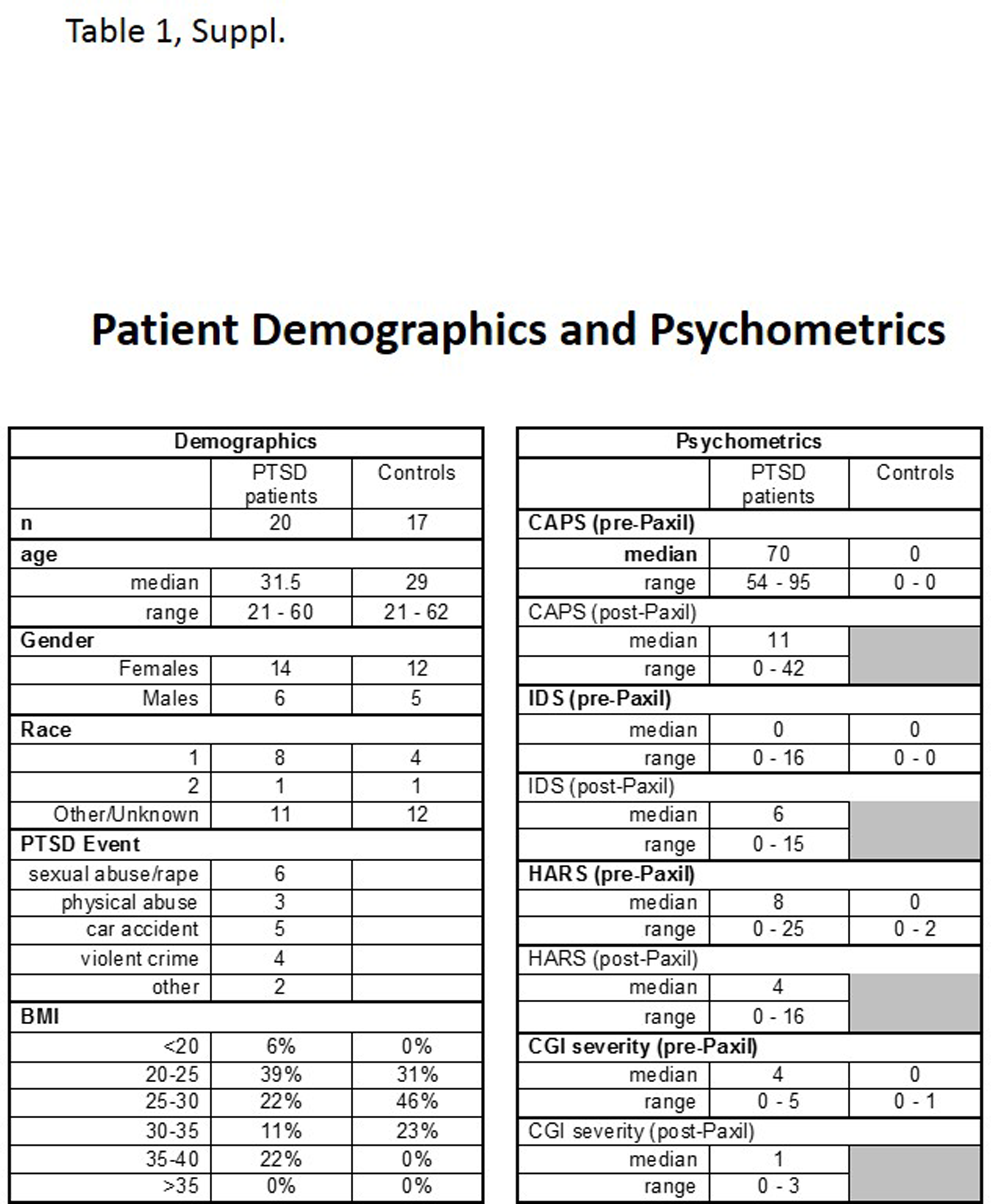

Supplement: Supplementary Table 1 [file tp2016285x2.tif]

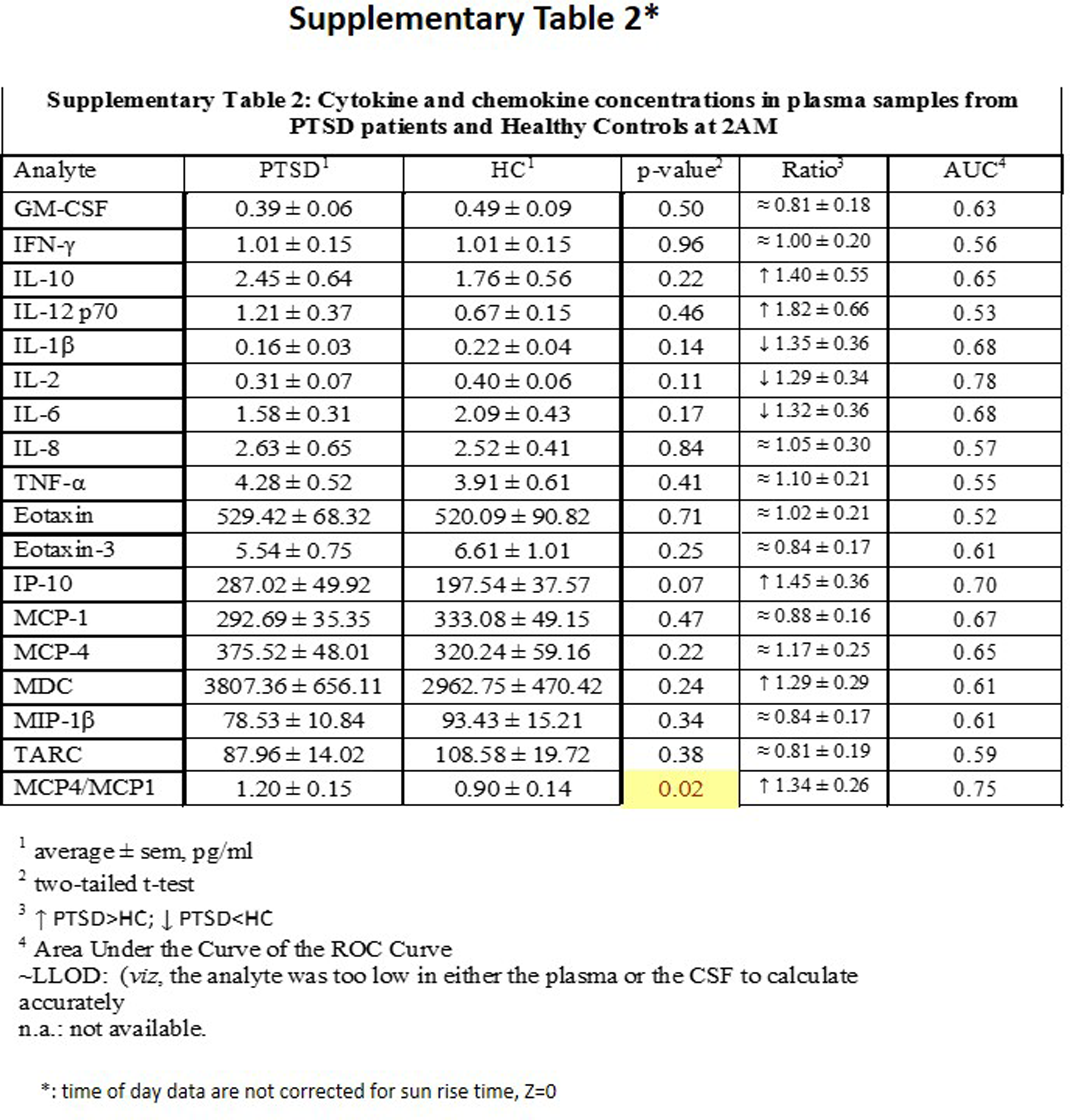

Supplement: Supplementary Table 2 [file tp2016285x3.tif]

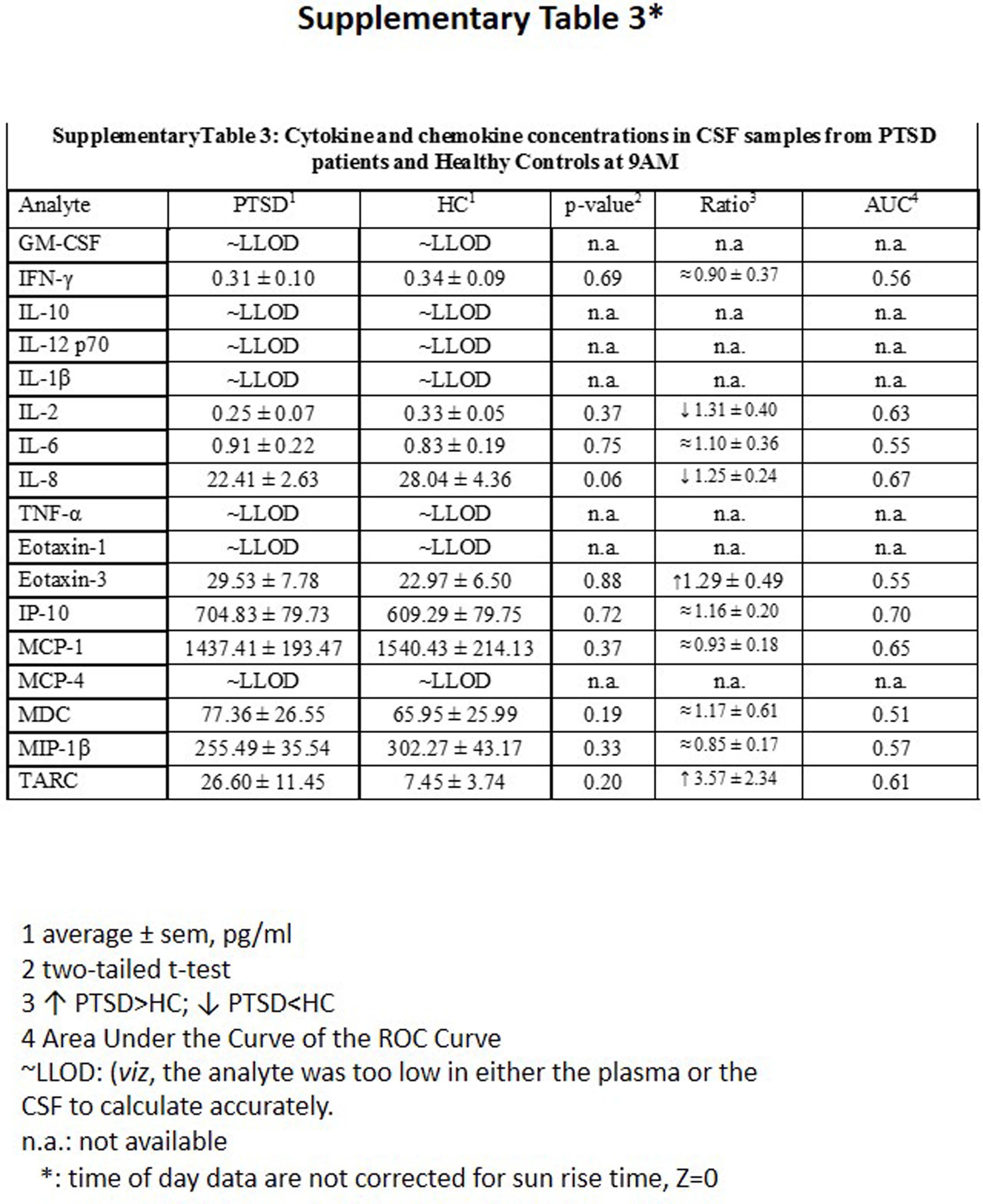

Supplement: Supplementary Table 3 [file tp2016285x4.tif]

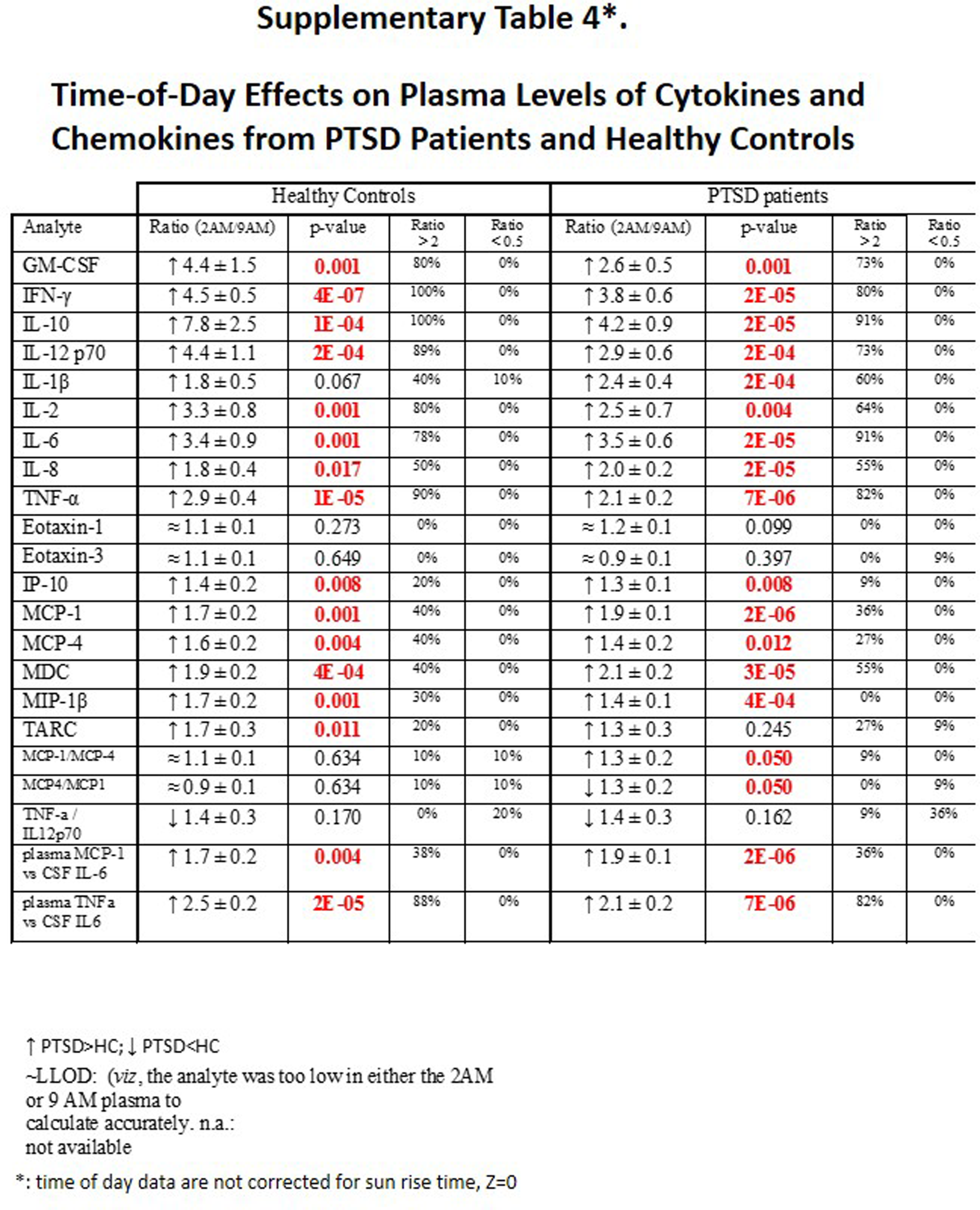

Supplement: Supplementary Table 4 [file tp2016285x5.tif]

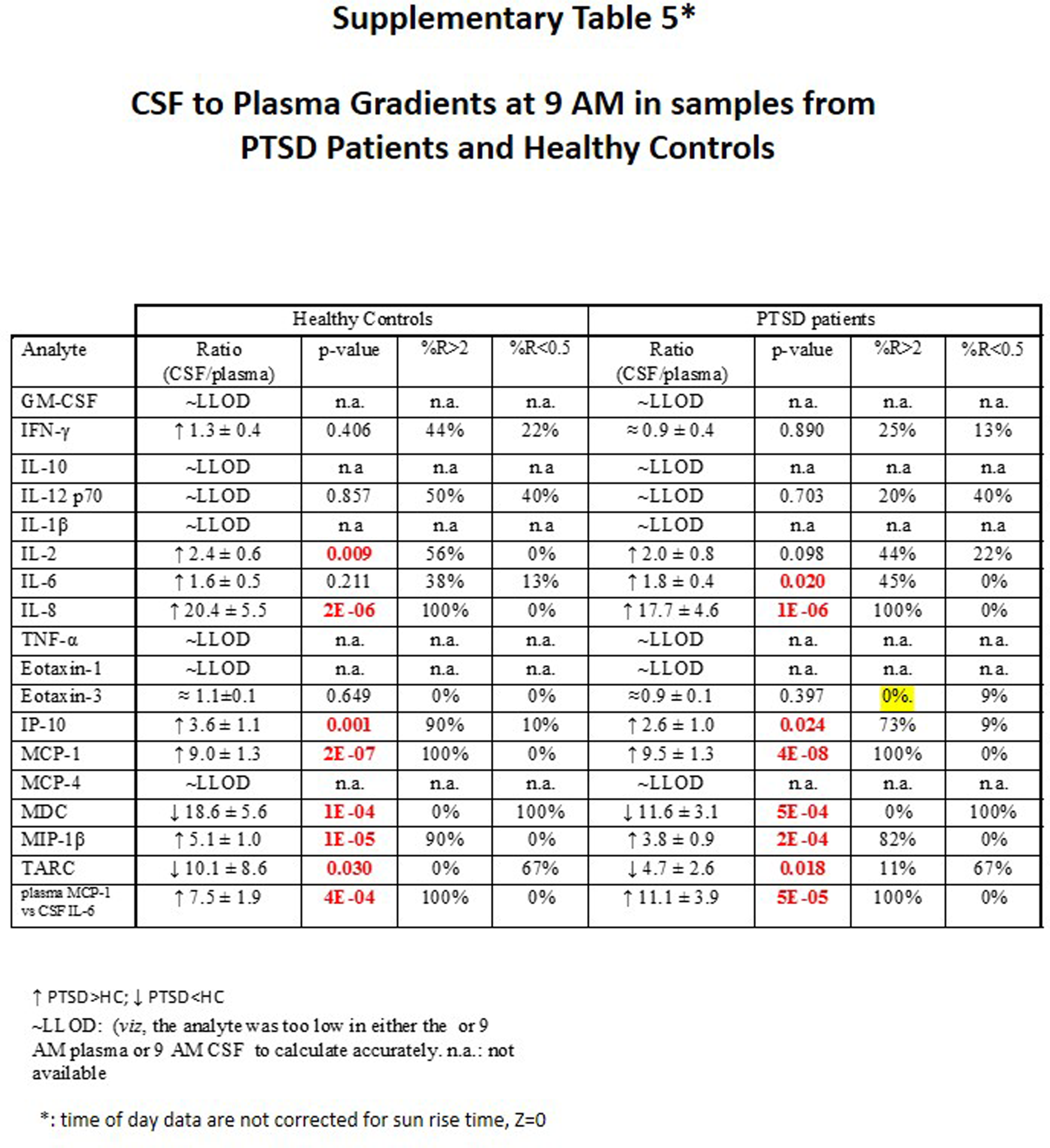

Supplement: Supplementary Table 5 [file tp2016285x6.tif]

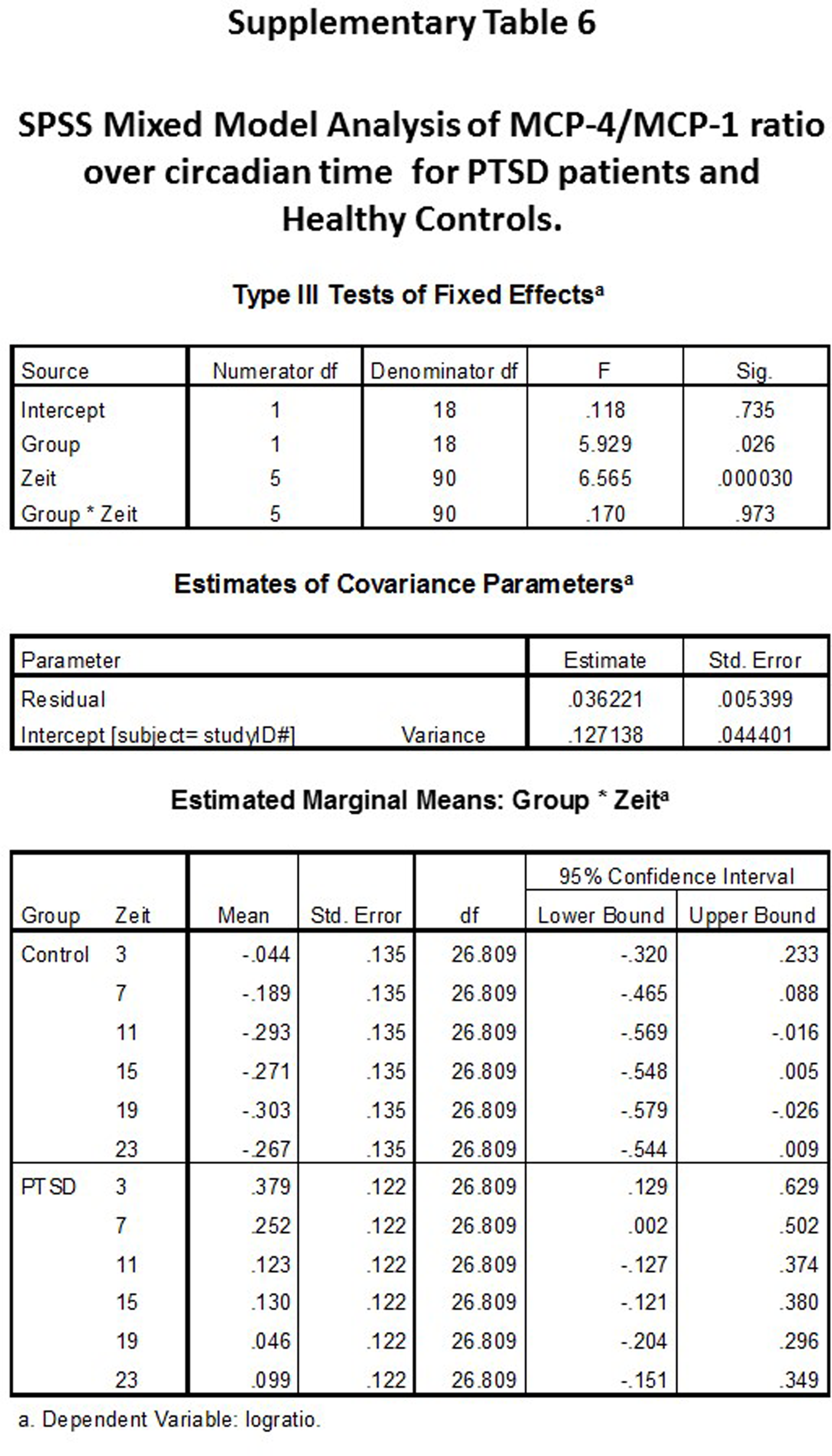

Supplement: Supplementary Table 6 [file tp2016285x7.tif]

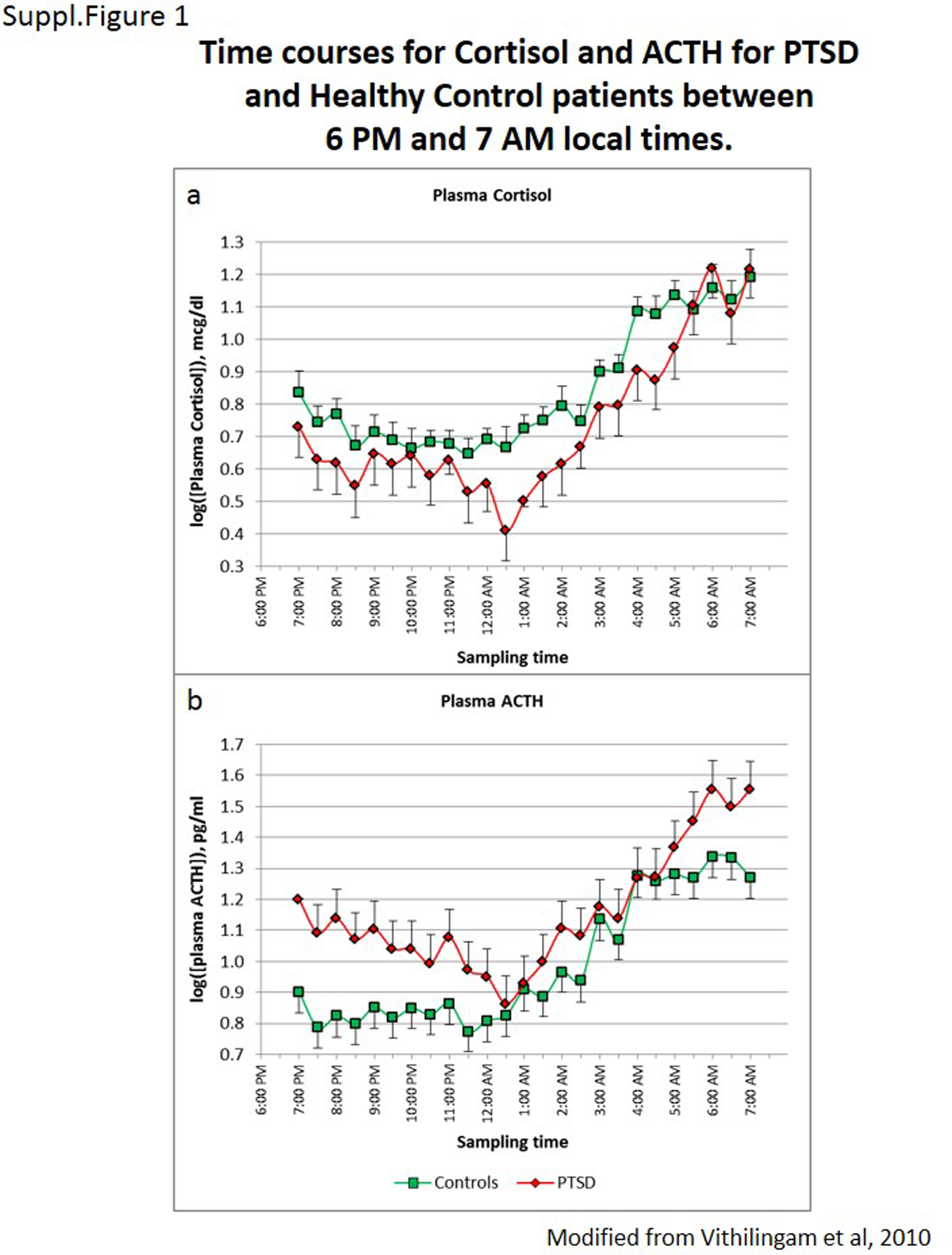

Supplement: Supplementary Figure 1 [file tp2016285x8.tif]

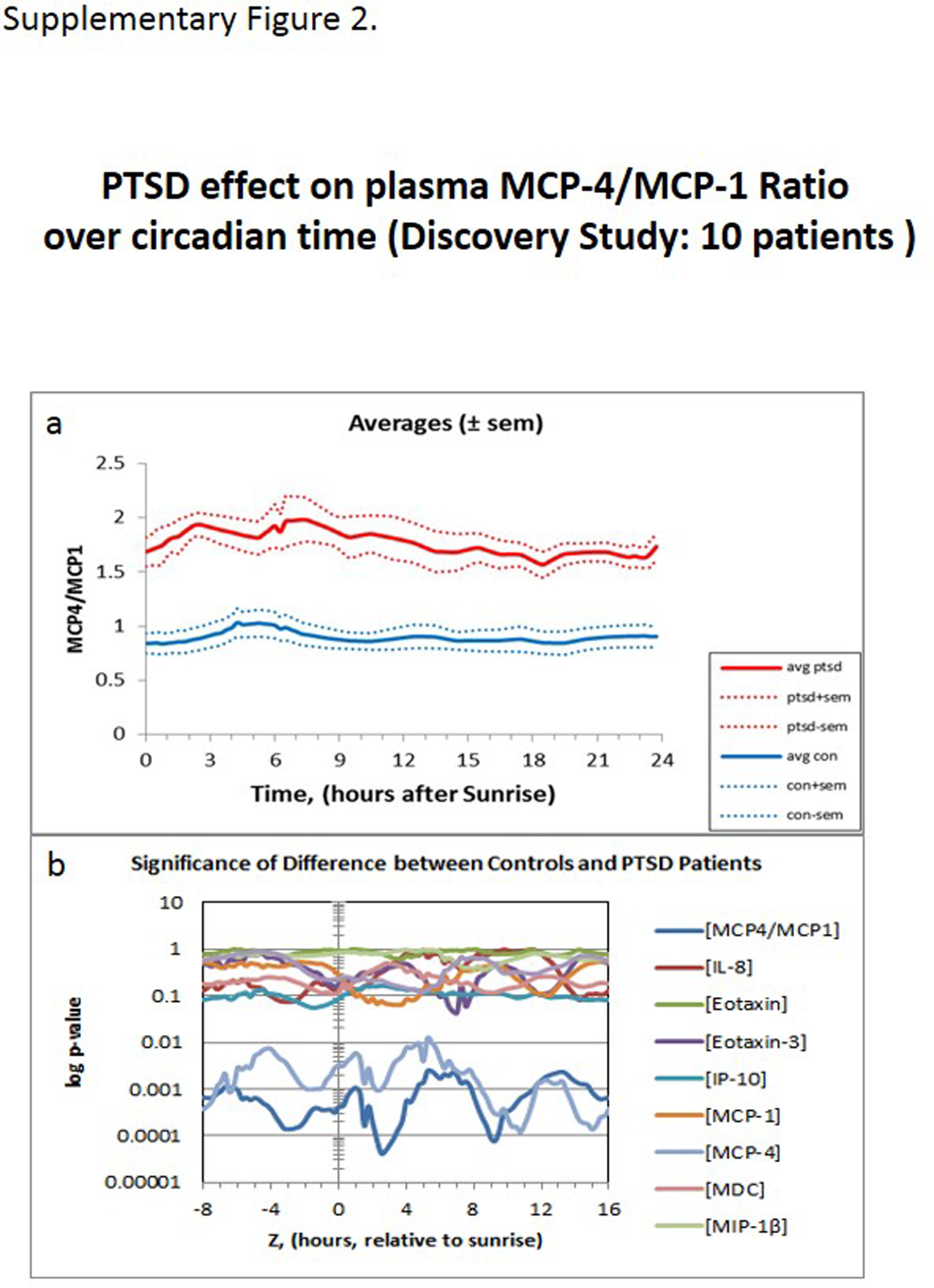

Supplement: Supplementary Figure 2 [file tp2016285x9.tif]

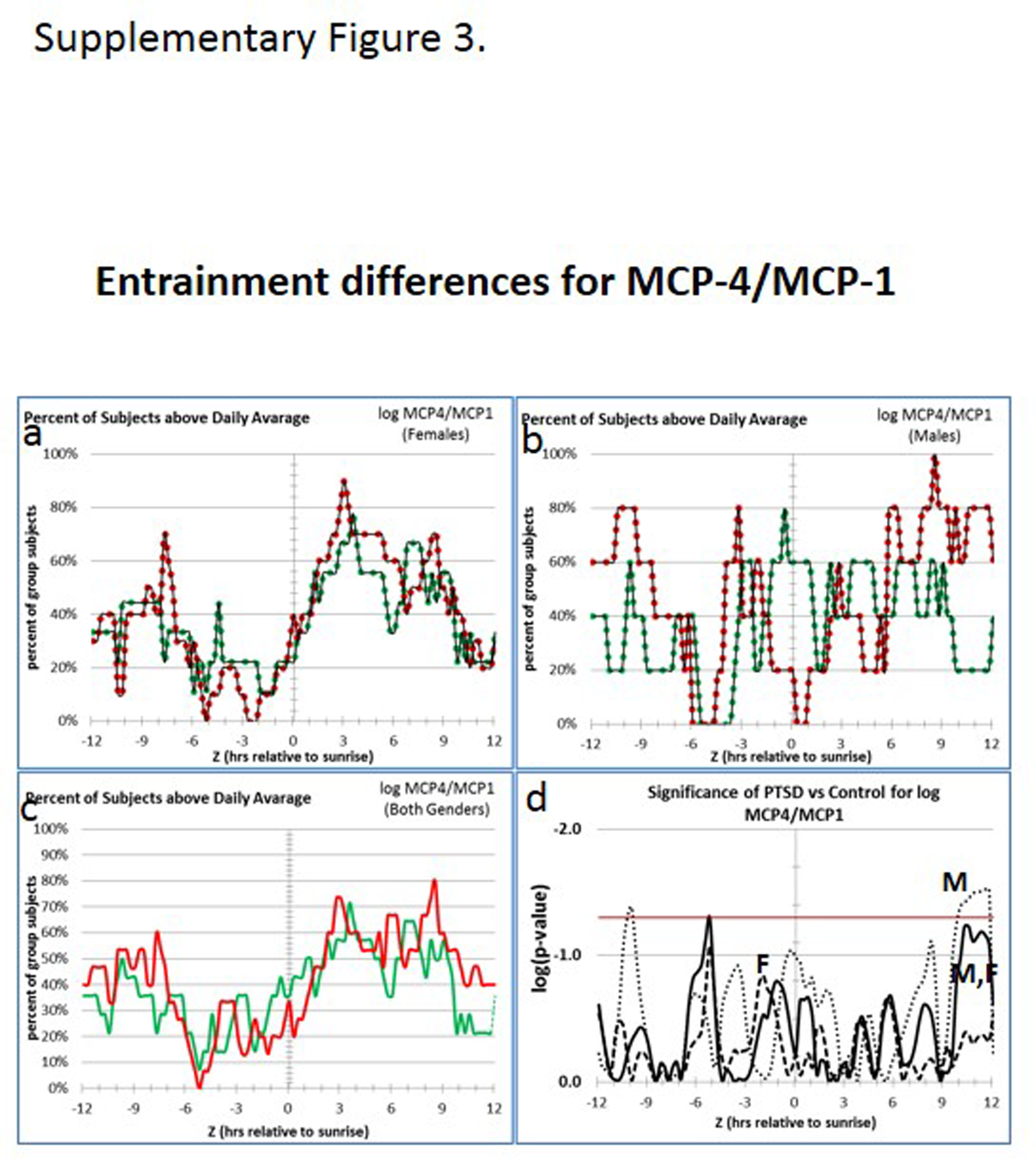

Supplement: Supplementary Figure 3 [file tp2016285x10.tif]

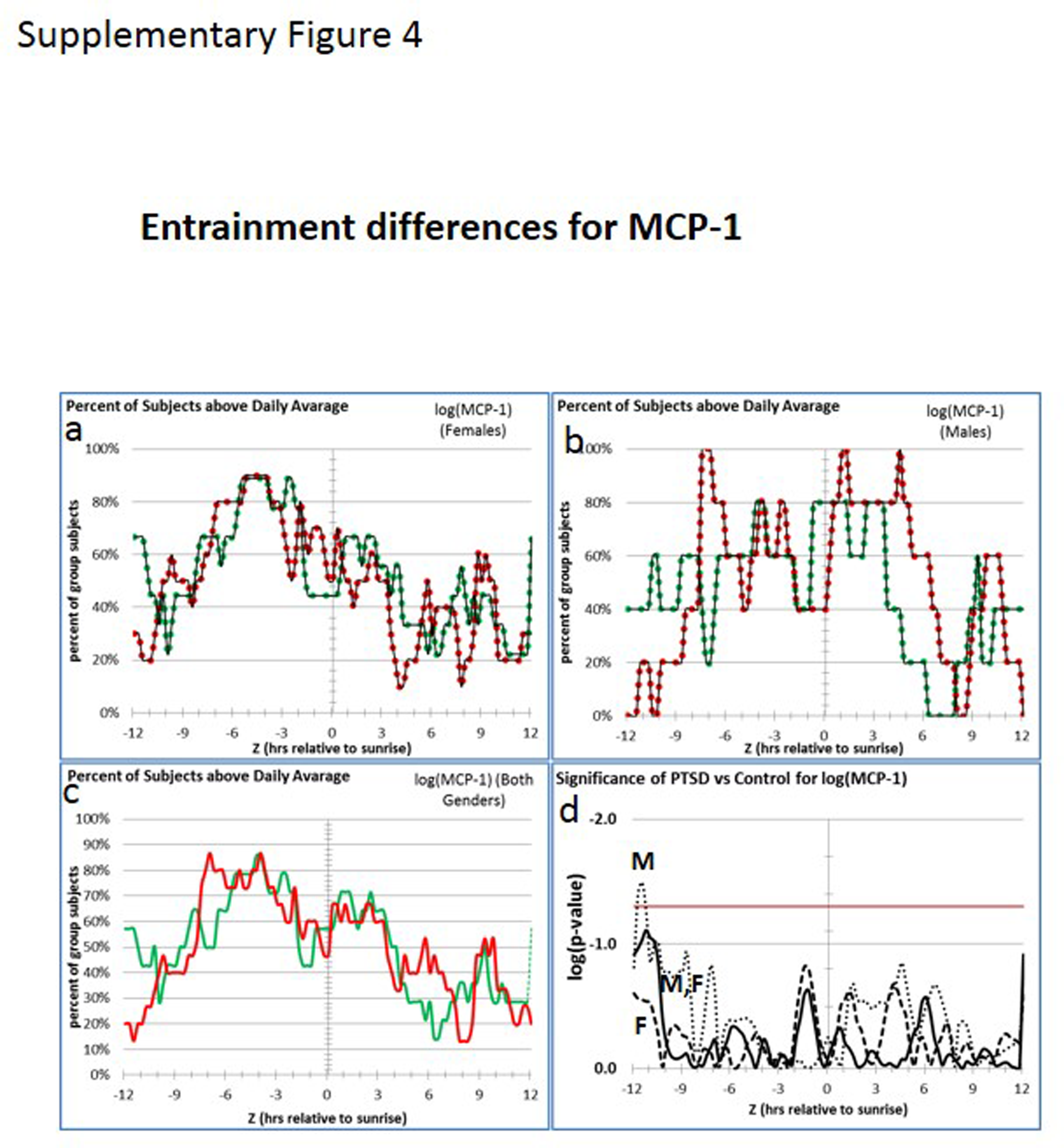

Supplement: Supplementary Figure 4 [file tp2016285x11.tif]

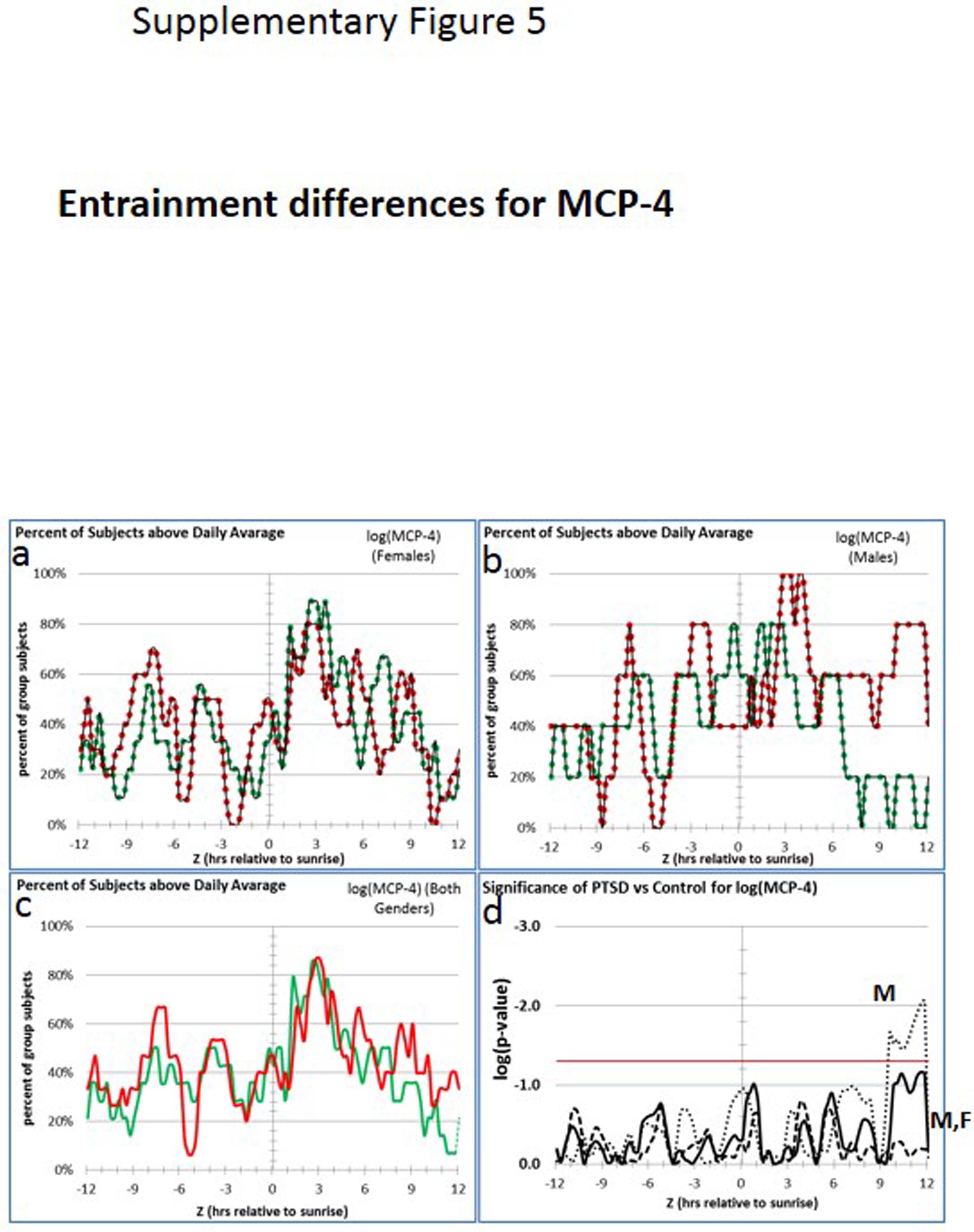

Supplement: Supplementary Figure 5 [file tp2016285x12.tif]

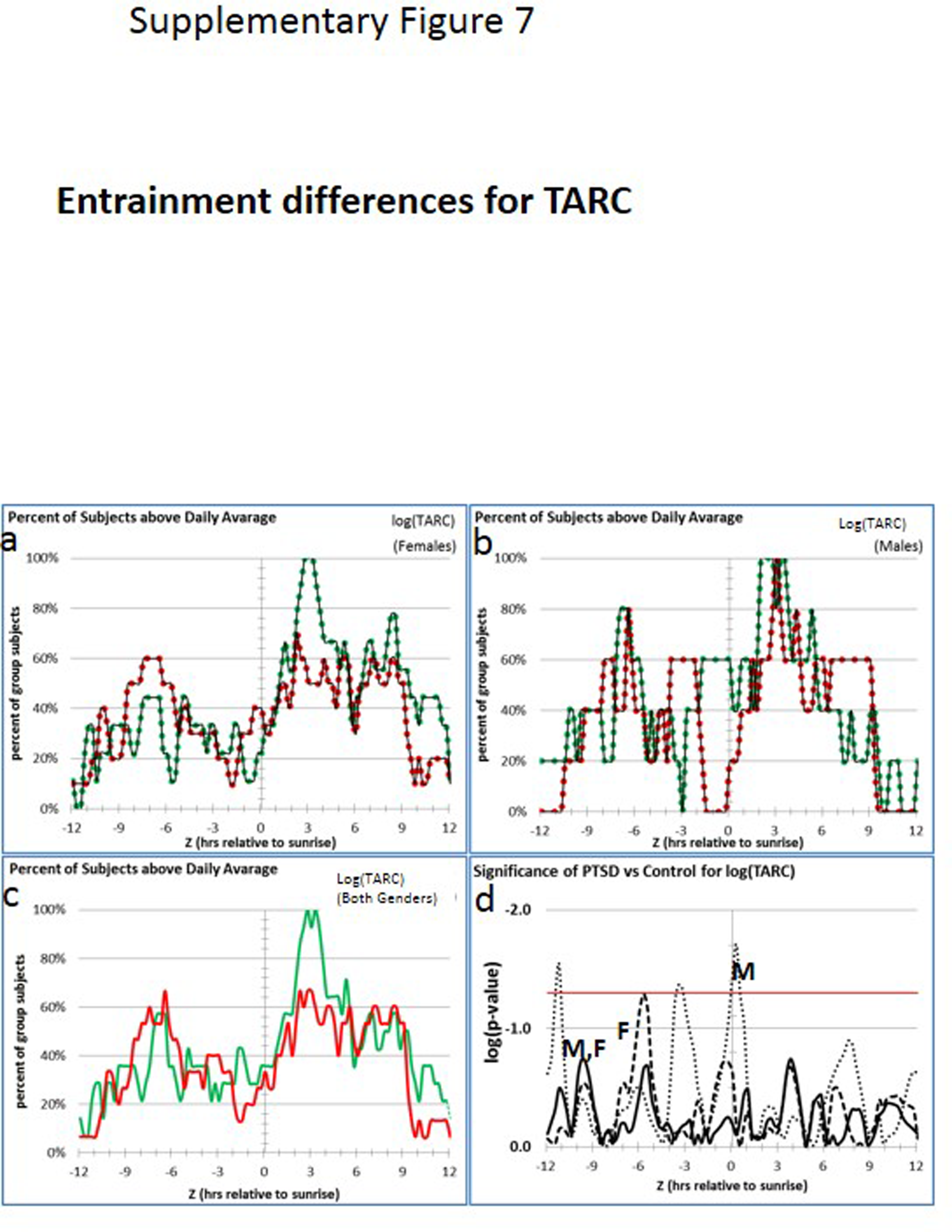

Supplement: Supplementary Figure 7 [file tp2016285x13.tif]

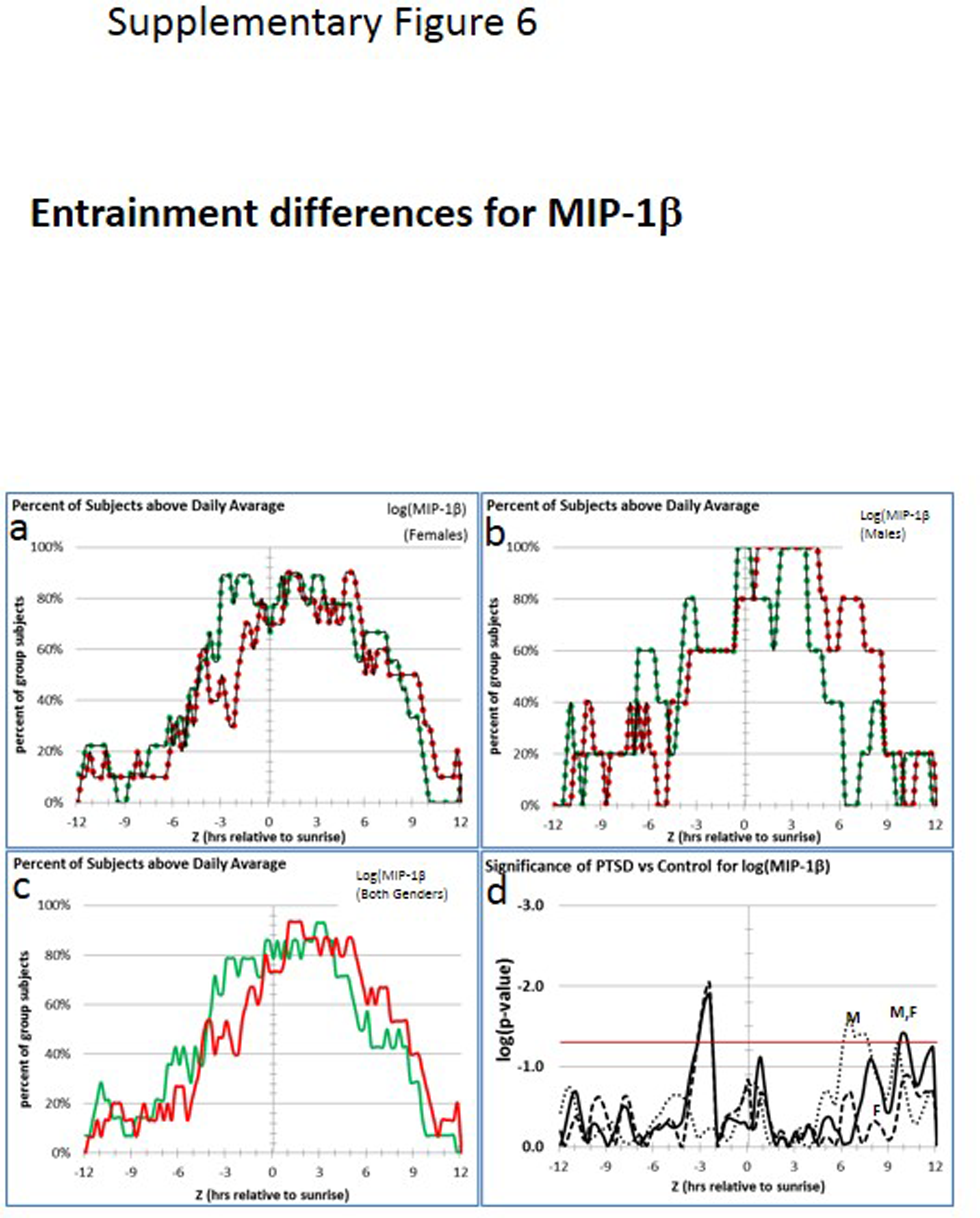

Supplement: Supplementary Figure 6 [file tp2016285x14.tif]

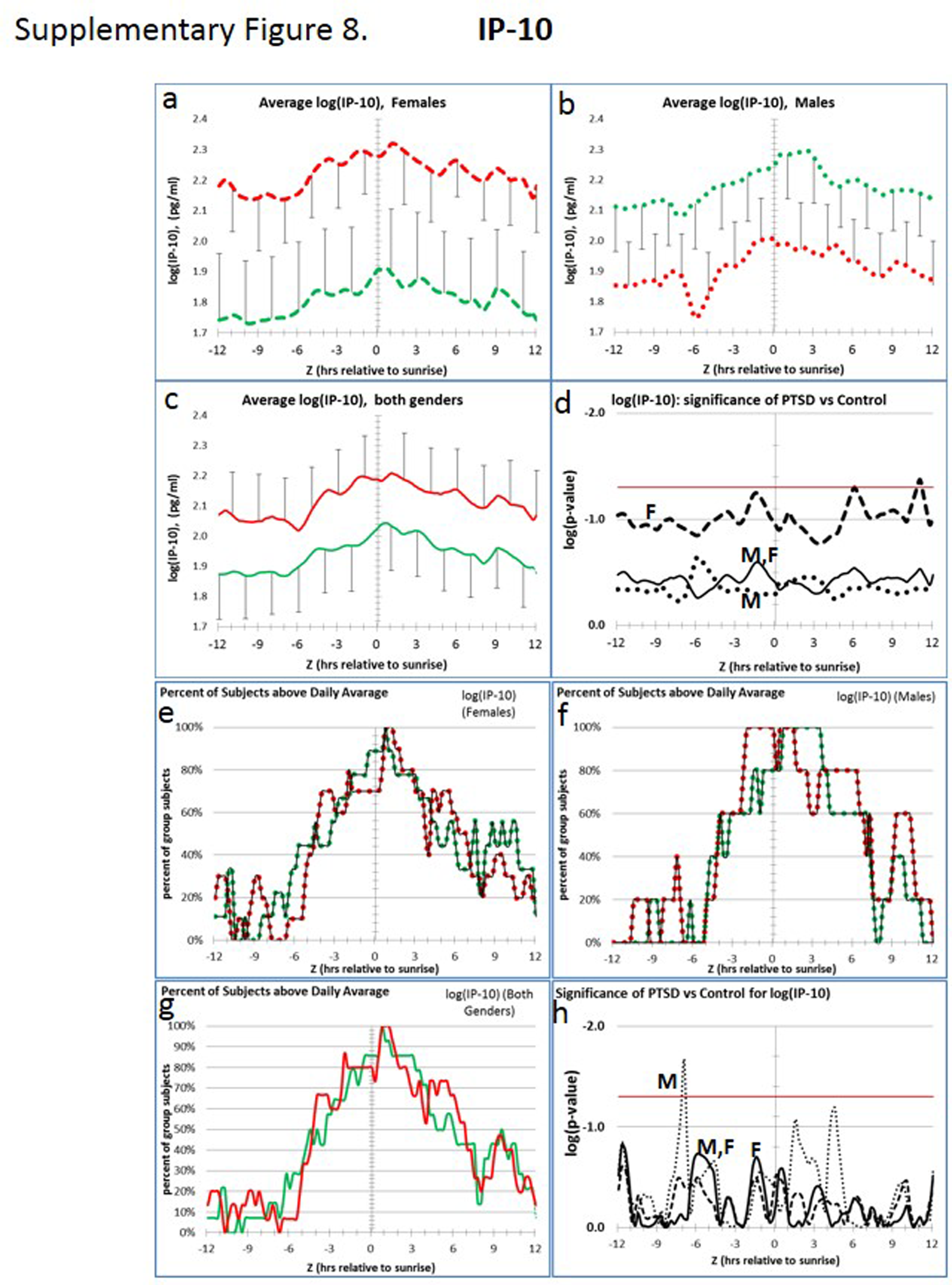

Supplement: Supplementary Figure 8 [file tp2016285x15.tif]

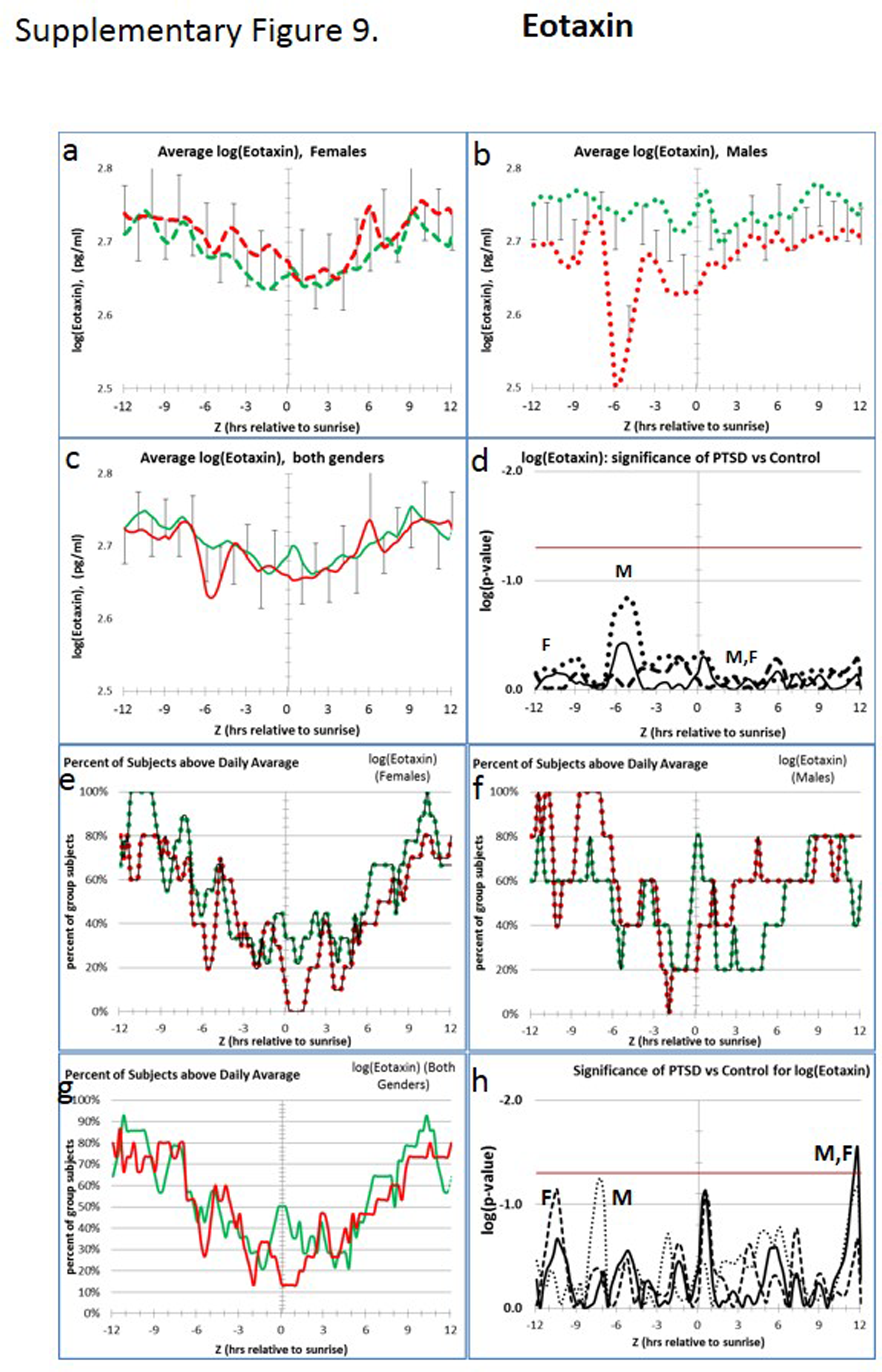

Supplement: Supplementary Figure 9 [file tp2016285x16.tif]

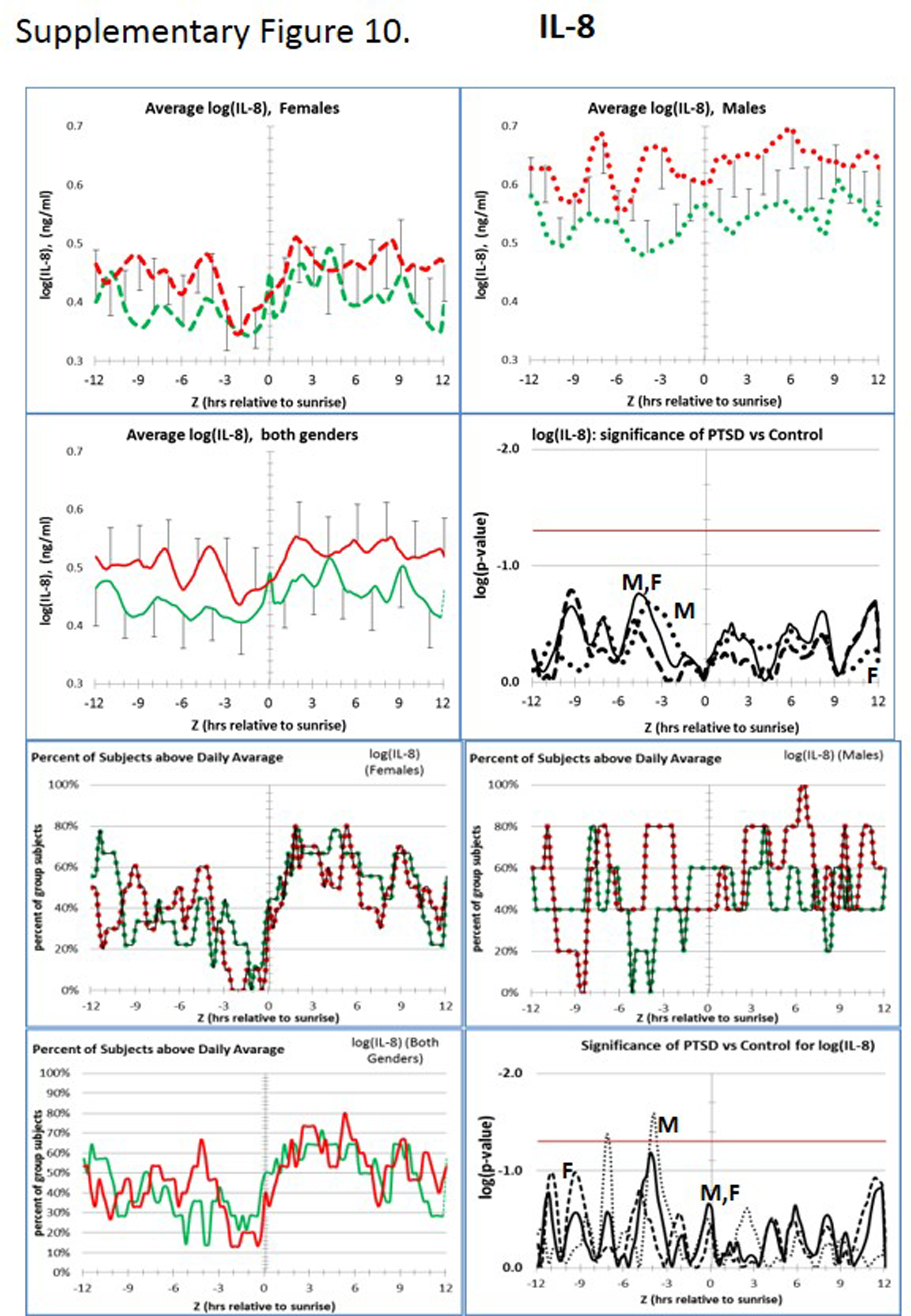

Supplement: Supplementary Figure 10 [file tp2016285x17.tif]

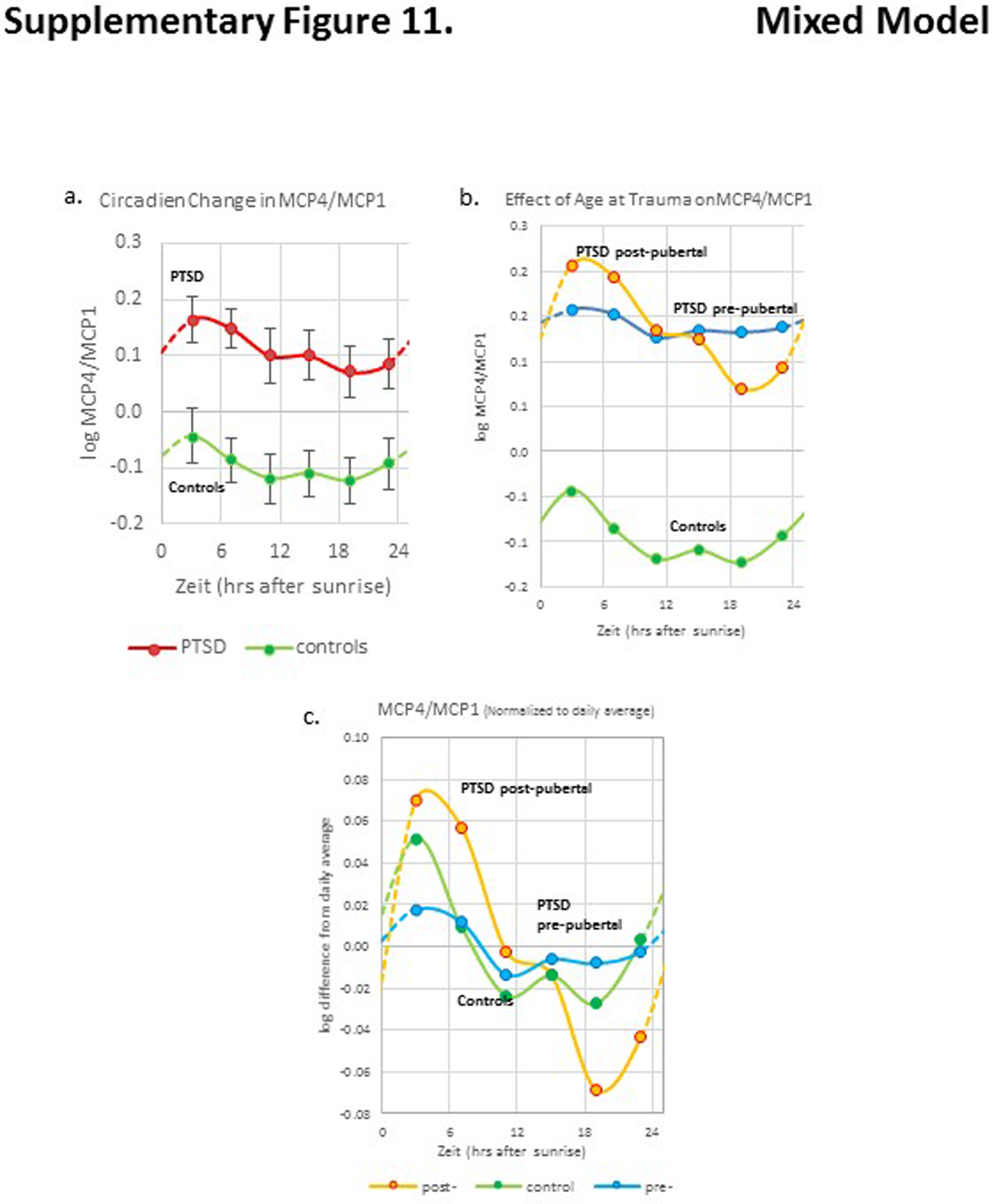

Supplement: Supplementary Figure 11 [file tp2016285x18.tif]
